# Assessment of bleeding in patients with disseminated intravascular coagulation after receiving surgery and recombinant human soluble thrombomodulin: A cohort study using a database

**DOI:** 10.1371/journal.pone.0205146

**Published:** 2018-10-08

**Authors:** Takuhiro Yamaguchi, Yukio Kitajima, Yasuhiro Miyauchi, Kazutoshi Izawa, Masakazu Tanaka, Masatada Hirata, Yasunari Sadatsuki, Yoshihiro Ogawa

**Affiliations:** 1 Division of Biostatistics, Tohoku University Graduate School of Medicine, Sendai, Japan; 2 Medical Affairs Division, CAC Croit Corporation, Chuo-ku, Tokyo, Japan; Maastricht University Medical Center, NETHERLANDS

## Abstract

We aimed to investigate the incidence of bleeding-related adverse events (AEs) among patients with disseminated intravascular coagulation (DIC) receiving recombinant thrombomodulin (rTM) and those receiving other DIC treatments, the incidence by type of surgery, and the incidence when either blood transfusion or a hemostatic procedure was administered to treat DIC. In this cohort study, data were obtained from a large medical database (22 centers in Japan). The primary endpoint was the incidence rate of bleeding-related AEs by type of surgery. The secondary endpoint was the incidence rate of bleeding-related AEs based on whether blood transfusion or a hemostatic procedure was administered after the day of DIC treatment. In total, 4234 propensity score-matched patients were included in the main analysis (2117 patients each in the rTM and non-rTM groups). In the rTM and non-rTM groups, respectively, the incidence of bleeding-related AEs was 18.8% and 24.8% (p <0.001; risk ratio [RR] 0.757, 95% confidence interval [CI] 0.674–0.849), among patients requiring any type of surgery; 15.0% and 19.5% (p = 0.0001; RR 0.769, 95% CI 0.673–0.879) in patients requiring blood transfusion or a hemostatic procedure after the day of DIC treatment; 10.2% and 11.6% (p = 0.4470; RR 0.879, 95% CI 0.630–1.226) in patients undergoing hepatic, biliary, or pancreatic surgery; 24.3% and 25.4% (p = 0.6439; RR 0.955, 95% CI 0.786–1.160) in patients undergoing gastrointestinal surgeries; and 18.5% and 30.1% (p = 0.0001; RR 0.614, 95% CI 0.481–0.782) in patients undergoing cardiac or cardiovascular surgery. Our findings suggest that rTM treatment for Japanese postsurgical patients who develop DIC was associated with significantly fewer bleeding-related AEs compared with those receiving other DIC treatments.

## Introduction

Disseminated intravascular coagulation (DIC) is a syndrome characterized by activation of blood coagulation, resulting in intravascular thrombin and fibrin generation, with thrombosis of small- to medium-sized vessels, consequently leading to organ dysfunction and severe bleeding [1]. A Japanese study based on a national administrative database reported that 34,711 DIC patients were referred to hospitals between 2010 and 2012 [2]. Among those with infectious diseases, the in-hospital mortality within 28 days was 27.7% in 2012 [2]. DIC can occur as a complication of infection, sepsis, solid cancers, hematological malignancies, obstetric diseases, trauma, aneurysms, and liver diseases.

DIC results from the interaction of two hemodynamic processes, hypercoagulation and hyperfibrinolysis. When hypercoagulation is predominant, as seen in patients with infection, particularly sepsis, the main sign is organ failure. This type of DIC is referred to as organ failure type, hypercoagulation predominance type, or hypofibrinolysis type. Bleeding is the primary symptom when hyperfibrinolysis is predominant (referred to as bleeding type or hyperfibrinolysis predominance type), as seen in patients with leukemia, obstetric diseases, or aortic aneurysms [3].

The prognosis of patients who present with DIC is extremely poor. In a previous report, a high resolution rate from DIC was observed in obstetric diseases and hematologic malignancies, but poor outcomes were observed in trauma/burn victims and those with infectious disease [4].

Current international and local treatment guidelines for DIC management [5–9] recommend the treatment of the underlying condition in patients with bleeding, organ failure, and non-symptomatic types of DIC. However, blood transfusions are needed in patients with massive bleeding secondary to DIC. Additional treatment aimed at normalizing/stabilizing the coagulation system abnormalities is sometimes required [3, 10]. This includes heparin or an antithrombin (AT-III) derivative, platelet concentrate, fresh frozen plasma, serine protease inhibitor, factor Xa and fibrinogen derivatives (hereafter referred to as “non-recombinant thrombomodulin [rTM] treatments”), and rTM.

rTM (Recomodulin^TM^) is composed of the active, extracellular domain of thrombomodulin and binds to thrombin to inactivate coagulation. The thrombin–rTM complex activates protein C to produce activated protein C, which inactivates factors VIIIa and factor Va in the presence of protein S, inhibiting further thrombin formation [11, 12]. rTM suppresses thrombin production at a concentration range that does not inhibit the blood-clotting activity of thrombin.

In a phase III study comparing the efficacy and safety of rTM to those of low-dose heparin for the treatment of DIC in 232 patients with hematologic malignancy or infection, rTM was found to be superior to heparin in terms of bleeding symptoms and DIC resolution [12]. Additionally, the incidence rates of bleeding-related adverse events (AEs) were lower with rTM compared with heparin. In terms of safety, rTM is superior to heparin, the current standard DIC therapy [12], as rTM does not increase the bleeding risk, despite resulting in sufficient anticoagulation. However, only a few postmarketing studies [13, 14] have investigated the incidence rates of bleeding-related AEs during rTM treatment in a real-world clinical setting.

For the present study, we hypothesized that rTM treatment for DIC after surgery does not increase the incidence of bleeding-related AEs compared with other DIC treatments. Thus, the main objective of this study was to determine whether there was a difference in the incidence of bleeding-related AEs between the rTM group and the group receiving other DIC treatments. For this purpose, we analyzed data from a large population obtained from the medical information database by Medical Data Vision Co., Ltd. (MDV). As surgery could influence the onset of bleeding-related AEs, we also investigated the incidence of such AEs by type of surgery (surgery of the liver, gallbladder, and pancreas; surgery related to other gastrointestinal organs; and surgery related to the heart and cardiovascular systems). Moreover, we investigated the incidence of bleeding-related AEs when blood transfusion or hemostatic procedures were administered to treat DIC. We showed that rTM treatment for Japanese postsurgical patients who develop DIC was associated with significantly fewer bleeding-related AEs compared with those receiving other DIC treatments, which confirmed our hypothesis.

## Materials and methods

### Database, study design, setting, and definitions

This was a cohort study using data from 22 centers in Japan obtained from a large medical database collected by MDV. The MDV medical information database is the largest medical information database in Japan, with more than 10 million registered patients (approximately 10% of the Japanese population)–a sample with a similar age distribution to the Japanese population; therefore, the data are considered highly generalizable. The database is based on Diagnosis Procedure Combination data and receipt data provided by acute-phase medical institutions in Japan and covers approximately 15% of all acute-phase medical institutions in Japan, as well as including information on medical treatments, drugs prescribed, and disease scale. As of the end of May 2017, approximately 18,630,000 pediatric and adult patients have been registered in the database.

All participants were Japanese. Participants were divided into two groups for comparison: patients with DIC receiving rTM (the rTM group) and patients with DIC treated with other agents (the non-rTM group). The study registration period was between 1 April 2010 and 31 August 2015.

Patients were considered to be exposed to rTM if the day of the initial administration of rTM was within 1 week after surgery. The initial administration date was defined as the date in which the patient received rTM for the first time. Data of patients who were initially administered rTM within 1 week after surgery were collected using the receipt code from the MDV medical information database. The follow-up period was defined as the time elapsed from the first day of the month when DIC treatment started up to the last day of the next month of surgery. A bleeding-related AE was defined based on the International Classification of Diseases version 10 (ICD-10), and an event was defined as when the diagnosed disease code corresponded to a defined list of bleeding AEs (**S1 Table**). Bleeding AEs included mild to severe events, such as bleeding from external and internal (organ) injuries and bleeding that caused inflammation, such as enteritis, hematoma, or purpura associated with bleeding, and blood disorders.

Approval of the study protocol was given by the Institutional Review Board of the Tohoku University Graduate School of Medicine (2017-1-537, approved on 25 September 2017). The need for informed consent was waived as this study analyzed anonymous patient data from a large database. This study is reported as per the Strengthening the Reporting of Observational Studies in Epidemiology guidelines (**S1 STROBE Checklist**).

### Participants

The target population of this study included patients who presented emergency rooms and who were then admitted to large medical institutions that were part of the database. The inclusion criteria were as follows: Inpatients diagnosed with DIC for the first time according to ICD-10 code D65 (disseminated intravascular coagulation [defibrination syndrome]); patients coded to have received DIC treatment with rTM and/or other agents on electronic medical and billing records; and patients coded to have received medical treatment defined as “surgery” on electronic medical and billing records (in this study, patients were classified into four groups as patients who received: 1) all types of surgery [including the following surgeries]; 2) hepatic, biliary, or pancreatic surgery; 3) gastrointestinal surgery; and 4) cardiac or cardiovascular surgery).

The exclusion criteria were as follows: Patients lacking a record of outcome data for hospital admission and/or discharge at the time of initial diagnosis of DIC; patients aged less than 18 years; patients with gynecologic/obstetric conditions (women who were pregnant or possibly pregnant); patients who died within 2 days after admission; patients who underwent surgery ≥1 month before the diagnosis of DIC; patients who did not undergo DIC treatment; patients who had received the initial dose of DIC treatment prior to the day of surgery or >1 week after surgery; patients lacking blood test data (peripheral blood); patients with intracranial hemorrhage, pulmonary hemorrhage, or gastrointestinal hemorrhage (contraindications for rTM treatment as stated in the Japanese product information) before DIC treatment; and patients who had received at least 1 dose of rTM but no initial prescription of rTM on the day of the surgery or within 1 week after surgery.

### Primary endpoint

The primary endpoint of this study was the incidence rate of bleeding-related AEs by type of surgery.

### Secondary endpoint

The secondary endpoint was the incidence rate of bleeding-related AEs based on whether or not blood transfusion or a hemostatic procedure was administered after the day of DIC treatment.

### Measurements and variables

Exposure was defined as the initial prescription of rTM or other treatments for DIC on the day of the surgery and within 1 week after surgery. The day of initial prescription was defined as the earliest date at which the patient was prescribed rTM or other non-rTM agents. The baseline characteristics recorded were sex, age, concomitant drugs, concomitant diseases, procedures required during hospitalization, blood or blood product transfusion, and category of the medical institution (based on the number of beds as hospital scale data).

The concomitant drugs allowed were as follows: catecholamine, antibiotics, antifungal drugs, other DIC treatments, steroids, neutrophil elastase inhibitor (sivelestat), and immunoglobulin. The non-rTM treatments allowed were AT-III derivative or heparin, platelet concentrate, fresh frozen plasma, serine protease inhibitor, and factor Xa and fibrinogen derivatives.

Concomitant diseases were defined as target diseases of the Charlson Comorbidity Index and included myocardial infarction, congestive heart failure, peripheral vascular disorder, cerebrovascular disorder, dementia, chronic pulmonary disease, collagen disease, peptic ulcer, mild hepatic disease, diabetes mellitus, hemiplegia, renal dysfunction, solid cancer/leukemia/lymphoma, moderate to severe renal dysfunction, metastatic solid tumor, and AIDS/HIV. Concomitant diseases were defined according to ICD-10 codes used in a previous study [15].

Procedures required during hospitalization were defined as ventilator, dialysis, extracorporeal membrane oxygenation, intraaortic balloon pumping, central venous catheterization, pleural effusion drainage, blood purification therapy, and blood test for the coagulation and fibrinolytic system. Blood transfusion was categorized as red blood cell transfusion and whole blood transfusion (irradiated leukocyte-reduced whole blood or leukocyte-reduced whole blood). Laboratory test values for platelet count (<80,000/mm^3^), creatinine, bilirubin, and fibrinogen/fibrin degradation products (FDP)/D-dimer were collected but not tested for intergroup comparison.

### Study size

In a survey of rTM use, it was reported that bleeding-related AEs were observed in 223/4062 patients (5.5%) [16]. Under the assumption that the incidence of bleeding-related AEs would be similar to the above in the rTM group and that the incidence of bleeding-related AEs was 6.1% (risk ratio: 0.9), 6.9% (risk ratio: 0.8), and 7.9% (risk ratio: 0.7) in the non-rTM group, the statistical power would be 13.4%, 47.5%, and 87.8%, respectively, for 2150 patients per group.

### Statistical analysis

Patient background characteristics are presented as n (%), mean ± standard deviation (SD), and median (minimum and maximum). Other variables are presented as risk ratios (point estimations and 95% confidence intervals [CI]). The risk ratio was log-transformed, and the Wald test was used to assess the significance of between-group differences. The present analysis was performed on selected patients from the database according to the inclusion and exclusion criteria. Propensity-matched rTM and Non-rTM groups were defined by the propensity score. By using background factors (sex, age, presence/absence of concomitant drugs, presence/absence of concomitant diseases, presence/absence of procedures, presence/absence of blood transfusion, and hospital category based on the number of beds [hospital scale data]), the propensity score was calculated using a logistic regression model. No laboratory parameters were used for propensity score calculation. The standard deviation of the logit of the propensity score was calculated.

For propensity score matching, we used the GREEDY algorithm (gmatch.sas available at http://www.mayo.edu/research/documents/gmatchsas/doc-10027248). One-to-one pair matching was conducted. rTM administration was set as the standard, and matching was conducted with the patients who were within the range of 0.25-fold of the standard deviation. SAS v 9.4 (SAS Institute, Cary, NC, USA) was used for conducting the statistical analyses.

## Results

### Participants

**Fig 1:**
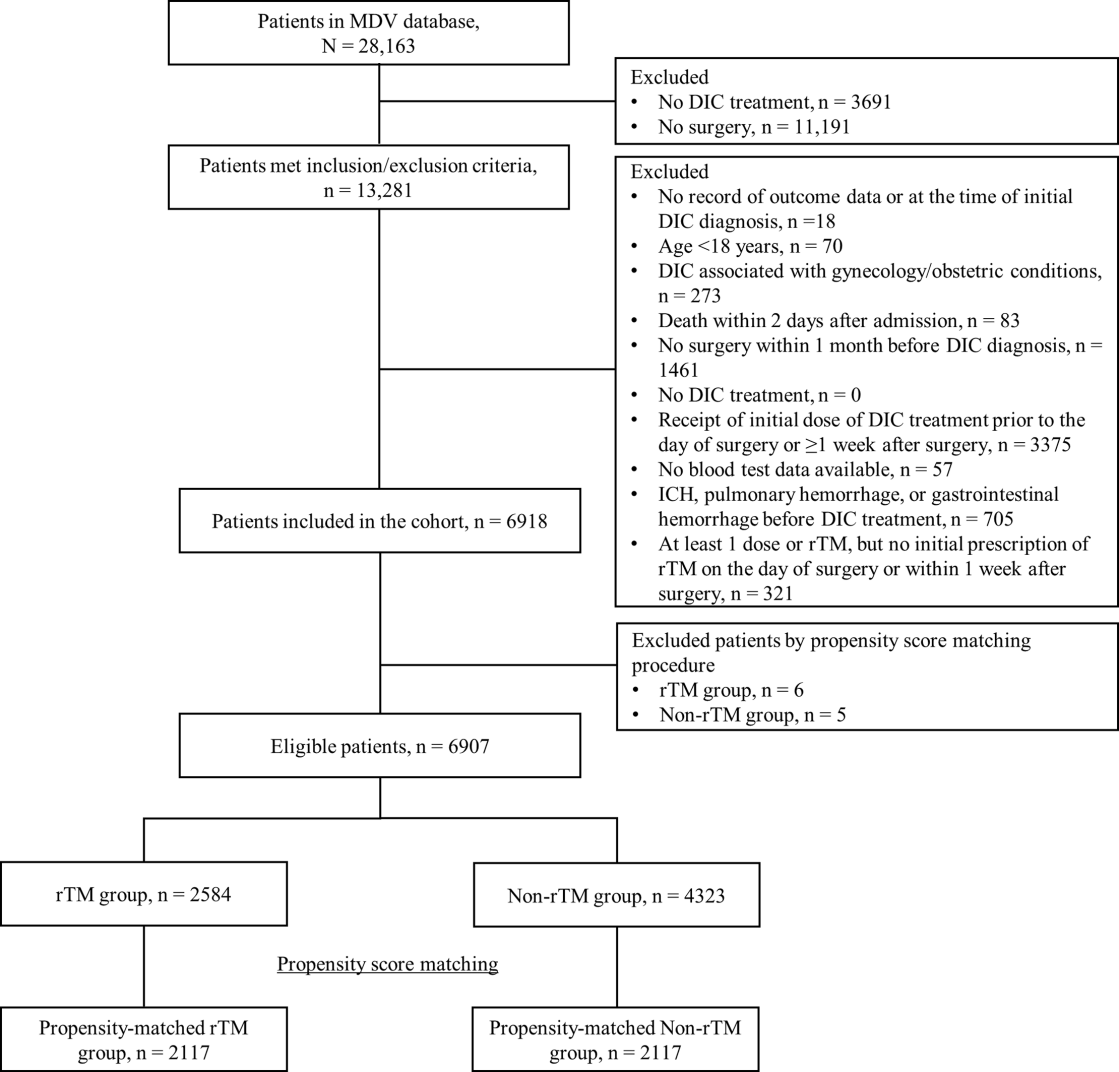
Flow diagram illustrating patient enrollment (all types of surgery). MDV, Medical Datavision; DIC, disseminated intravascular coagulation; rTM, recombinant thrombomodulin; ICH, intracranial hemorrhage.

**Fig 1** shows the patient disposition and the number of patients analyzed for propensity score matching and included in the analysis set for each treatment group.

Of the 28,163 patients identified in the database, 6918 were included in the cohort after applying the inclusion and exclusion criteria. The propensity score was calculated for 2584 and 4323 patients in each group. After propensity score matching, a total of 4234 patients were included in the main analysis: 2117 patients in the rTM group and 2117 patients in the non-rTM group.

Of the 4323 patients who underwent surgery of any type, 1136 patients underwent hepatic, biliary, or pancreatic surgery; 1228 patients underwent other gastrointestinal surgery; and 878 patients underwent cardiac or cardiovascular surgery. Patient disposition by type of surgery is shown in **S1–S3 Figs**.

### Baseline characteristics

Patient baseline demographic and clinical characteristics are shown in **Table 1.**

**Table 1:** Demographic and clinical baseline characteristics.

Item	Classification		Before matching			After matching		
			rTM group	non-rTM group	Standardized difference %	rTM group	non-rTM group	Standardized difference %
			N = 2590	N = 4328		N = 2117	N = 2117	
Sex, n (%)	Male		1350 (52.1)	2589 (59.8)	15.6	1125 (53.1)	1097 (51.8)	2.6
	Female		1240 (47.9)	1739 (40.2)	-	992 (46.9)	1020 (48.2)	-
Age, years	n		2590	4328	-	2117	2117	-
	Median (Minimum/ maximum)		76.0 (18/102)	74.0 (18/103)	-	76.0 (18/102)	76.0 (19/103)	-
Concomitant drugs, n (%)	Catecholamine	No	981 (37.9)	1840 (42.5)	9.5	886 (41.9)	912 (43.1)	2.5
		Yes	1609 (62.1)	2488 (57.5)	-	1231 (58.1)	1205 (56.9)	-
	Antibiotics	No	62 (2.4)	267 (6.2)	18.7	60 (2.8)	53 (2.5)	2.1
		Yes	2528 (97.6)	4061 (93.8)	-	2057 (97.2)	2064 (97.5)	-
	Antifungals	No	2462 (95.1)	4252 (98.2)	17.8	2041 (96.4)	2051 (96.9)	2.6
		Yes	128 (4.9)	76 (1.8)	-	76 (3.6)	66 (3.1)	-
	Other drugs for DIC treatment	No	737(28.5)	0(0.0)	89.2	690 (32.6)	0 (0.0)	98.3
		Yes	1853 (71.5)	4328 (100.0)	-	1427 (67.4)	2117(100.0)	-
	Steroids	No	1969 (76.0)	3285 (75.9)	0.3	1647 (77.8)	1676 (79.2)	3.3
		Yes	621 (24.0)	1043 (24.1)	-	470 (22.2)	441 (20.8)	-
	Neutrophil elastase inhibitors (Sivelestat)	No	2208 (85.3)	3975 (91.8)	20.8	1883 (88.9)	1884 (89.0)	0.2
		Yes	382 (14.7)	353(8.2)	-	234 (11.1)	233 (11.0)	-
	Immunoglobulins	No	1787 (69.0)	3765 (87.0)	44.5	1603 (75.7)	1624 (76.7)	2.3
		Yes	803 (31.0)	563 (13.0)	-	514 (24.3)	493 (23.3)	-
Complications, n (%)	Myocardial infarction	No	2532 (97.8)	4121 (95.2)	13.9	2063 (97.4)	2065 (97.5)	0.6
		Yes	58 (2.2)	207 (4.8)	-	54 (2.6)	52 (2.5)	-
	Congestive heart failure	No	2326 (89.8)	3631 (83.9)	17.6	1890 (89.3)	1886 (89.1)	0.6
		Yes	264 (10.2)	697 (16.1)	-	227 (10.7)	231 (10.9)	-
	Peripheral vascular disease	No	2513 (97.0)	3993 (92.3)	21.3	2049 (96.8)	2047 (96.7)	0.5
		Yes	77 (3.0)	335 (7.7)	-	68 (3.2)	70 (3.3)	-
	Cerebral vascular disease	No	2432 (93.9)	4024 (93.0)	3.7	1980 (93.5)	1973 (93.2)	1.3
		Yes	158 (6.1)	304 (7.0)	-	137 (6.5)	144 (6.8)	-
	Dementia	No	2526 (97.5)	4260 (98.4)	6.4	2064 (97.5)	2064 (97.5)	0.0
		Yes	64 (2.5)	68 (1.6)	-	53 (2.5)	53 (2.5)	-
	Chronic lung disease	No	2508 (96.8)	4144 (95.7)	5.7	2042 (96.5)	2046 (96.6)	1.0
		Yes	82 (3.2)	184 (4.3)	-	75 (3.5)	71 (3.4)	-
	Collagen disease	No	2558 (98.8)	4271 (98.7)	0.7	2090 (98.7)	2086 (98.5)	1.6
		Yes	32 (1.2)	57 (1.3)	-	27 (1.3)	31 (1.5)	-
	Peptic ulcer	No	2385 (92.1)	3920 (90.6)	5.4	1938 (91.5)	1954 (92.3)	2.8
		Yes	205 (7.9)	408 (9.4)	-	179 (8.5)	163 (7.7)	-
	Mild liver disease^a^	No	2448 (94.5)	3856 (89.1)	19.9	1988 (93.9)	2007 (94.8)	3.9
		Yes	142 (5.5)	472 (10.9)	-	129 (6.1)	110 (5.2)	-
	Diabetes	No	2264 (87.4)	3693 (85.3)	6.1	1830 (86.4)	1833 (86.6)	0.4
		Yes	326 (12.6)	635 (14.7)	-	287 (13.6)	284 (13.4)	-
	Hemiplegia	No	2583 (99.7)	4315 (99.7)	0.6	2111 (99.7)	2113 (99.8)	1.9
		Yes	7 (0.3)	13(0.3)	-	6 (0.3)	4 (0.2)	-
	Renal dysfunction	No	2403 (92.8)	4074 (94.1)	5.5	1981 (93.6)	1982 (93.6)	0.2
		Yes	187 (7.2)	254 (5.9)	-	136 (6.4)	135 (6.4)	-
	Diabetes mellitus with chronic complications	No	2514 (97.1)	4211 (97.3)	1.4	2057 (97.2)	2059 (97.3)	0.6
		Yes	76 (2.9)	117 (2.7)	-	60 (2.8)	58 (2.7)	-
	Solid cancer, leukemia, lymphoma	No	2099 (81.0)	3268 (75.5)	13.5	1672 (79.0)	1700 (80.3)	3.3
		Yes	491 (19.0)	1060 (24.5)	-	445 (21.0)	417 (19.7)	-
	Moderate to high liver dysfunction	No	2555 (98.6)	4195 (96.9)	11.7	2083 (98.4)	2091 (98.8)	3.2
		Yes	35(1.4)	133 (3.1)	-	34 (1.6)	26 (1.2)	-
	Metastatic solid tumors	No	2445 (94.4)	3997 (92.4)	8.2	1987 (93.9)	1993 (94.1)	1.2
		Yes	145 (5.6)	331 (7.6)	-	130 (6.1)	124 (5.9)	-
	AIDS/HIV	No	2588 (99.9)	4327 (100.0)	2.4	2116 (100.0)	2117 (100.0)	3.1
		Yes	2 (0.1)	1 (0.0)	-	1 (0.0)	0 (0.0)	-
Treatments, n (%)	Ventilators	No	1831 (70.7)	3710 (85.7)	37.0	1675 (79.1)	1705 (80.5)	3.5
		Yes	759 (29.3)	618 (14.3)	-	442 (20.9)	412 (19.5)	-
	Dialysis	No	2157 (83.3)	4047 (93.5)	32.3	1891 (89.3)	1901 (89.8)	1.5
		Yes	433 (16.7)	281 (6.5)	-	226 (10.7)	216 (10.2)	-
	Extracorporeal membrane oxygenation	No	2581 (99.7)	4054 (93.7)	33.8	2108 (99.6)	2112 (99.8)	3.3
		Yes	9 (0.3)	274 (6.3)	-	9 (0.4)	5 (0.2)	-
	Intra-aortic balloon pumping	No	2555 (98.6)	4232 (97.8)	6.6	2083 (98.4)	2091 (98.8)	3.2
		Yes	35 (1.4)	96 (2.2)	-	34 (1.6)	26 (1.2)	-
	Central venous catheterization	No	2578 (99.5)	4298 (99.3)	3.0	2105 (99.4)	2107 (99.5)	1.3
		Yes	12 (0.5)	30 (0.7)	-	12 (0.6)	10 (0.5)	-
	Pleural effusion	No	1681 (64.9)	3766 (87.0)	53.6	1586 (74.9)	1635 (77.2)	5.4
		Yes	909 (35.1)	562 (13.0)	-	531 (25.1)	482 (22.8)	-
	Blood purification therapy	No	2157 (83.3)	4047 (93.5)	32.3	1891 (89.3)	1901 (89.8)	1.5
		Yes	433 (16.7)	281 (6.5)	-	226 (10.7)	216 (10.2)	-
	Coagulation blood test	No	89 (3.4)	623 (14.4)	39.2	88 (4.2)	71 (3.4)	4.2
		Yes	2501 (96.6)	3705 (85.6)	-	2029 (95.8)	2046 (96.6)	-
Transfusion, n (%)	Red blood cell transfusion	No	1852 (71.5)	2748 (63.5)	17.2	1550 (73.2)	1563 (73.8)	1.4
		Yes	738 (28.5)	1580 (36.5)	-	567 (26.8)	554 (26.2)	-
	Whole blood transfusion	No	2590 (100.0)	4328 (100.0)	-	2117 (100.0)	2117 (100.0)	-
		Yes	0 (0.0)	0 (0.0)	-	0 (0.0)	0 (0.0)	-
Number of beds in medical facilities, n (%)	<200 beds		53 (2.0)	222 (5.1)	16.6	44 (2.1)	97 (4.6)	14.0
	≥200, <500		1492 (57.6)	2246 (51.9)	11.5	1237 (58.4)	1138 (53.8)	9.4
	≥500		1045 (40.3)	1860 (43.0)	5.3	836 (39.5)	882 (41.7)	4.4
Platelets (average^b^)	n		175	203	-	141	96	-
	Median		12.20	14.70	-	12.10	12.10	-
Creatinine (average^b^)	n		174	203	-	140	96	-
	Median		1.060	0.850	-	1.065	0.825	-
Total bilirubin (average^b^)	n		163	197	-	129	95	-
	Median		0.840	0.800	-	0.840	0.810	-
Direct bilirubin (average^b^)	n		92	110	-	73	56	-
	Median		0.310	0.375	-	0.300	0.500	-
FDP/D-dimer (average^b^)	n		60	56	-	43	26	-
	Median		8.717	8.150	-	8.833	7.600	-
Platelets (minimum^c^)	n		175	203	-	141	96	-
	Median		11.80	14.30	-	11.80	11.90	-
Creatinine (minimum^c^)	n		174	203	-	140	96	-
	Median		1.025	0.850	-	1.030	0.825	-
Total bilirubin (minimum^c^)	n		163	197	-	129	95	-
	Median		0.800	0.800	-	0.800	0.810	-
Direct bilirubin (minimum^c^)	n		92	110	-	73	56	-
	Median		0.310	0.375	-	0.300	0.500	-
FDP/D-dimer (minimum^c^)	n		60	56	-	43	26	-
	Median		8.550	8.150	-	8.600	7.600	-
Platelets (maximum^d^)	n		175	203		141	96	
	Median		12.30	15.00	-	12.10	12.10	-
Creatinine (maximum^d^)	n		174	203	-	140	96	-
	Median		1.065	0.850	-	1.075	0.825	-
Total bilirubin (maximum^d^)	n		163	197	-	129	95	-
	Median		0.870	0.800	-	0.840	0.810	-
Direct bilirubin (maximum^d^)	n		92	110	-	73	56	-
	Median		0.310	0.375	-	0.300	0.500	-
FDP/D-dimer (maximum^d^)	n		60	56	-	43	26	-
	Median		8.800	8.150	-	9.000	7.600	-

rTM, recombinant thrombomodulin; DIC, disseminated intravascular coagulation; FDP, fibrinogen/fibrin degradation products. The total number of patients in each group (2,590 and 4,328 in the rTM and non-rTM group, respectively) shown in Table 1.

^a^Includes severe liver disease (e.g., liver cirrhosis) as defined in a previous study [14].

^b^When there were repeated test results, we adopted the average.

^c^When there were repeated test results, we adopted the minimum.

^d^When there were repeated test results, we adopted the maximum.

The mean (± SD) age was 74.1 ± 12.8 years in the rTM group and 72.3 ± 13.2 years in the non-rTM group before matching (standardized difference = 13.6%), and 73.8 ± 13.0 years in the rTM group and 74.1 ± 13.0 years in the non-rTM group (standardized difference = 2.5%) after matching. Of the enrolled patients, 52.1% (n = 1350) in the rTM group and 59.8% (n = 2589) in the non-rTM group were males before matching. After matching, the proportion of male patients was 53.1% (n = 1125) and 51.8% (n = 1097) in the rTM and non-rTM groups, respectively. The usage ratio of non-rTM treatments is shown in **S2 Table**.

The standardized difference changes before and after matching in Table 1 showed that bias was adjusted. Patient baseline demographic and clinical characteristics by type of surgery before and after propensity score matching are shown in the **S3–S5 Tables**.

Regarding the propensity score, the logistic model was a poor fit for the observed data, based on the significant Hosmer–Lemeshow test result (χ^2^ = 32.6536, flexibility = 8, *p* < 0.0001). The ability of the propensity score to control for confounding was evaluated by the c-statistic (area under the receiver operating characteristic curve, 0.81), which showed moderate accuracy (any surgery [0.796], hepatic, biliary or pancreatic surgery [0.765]; other gastrointestinal surgery [0.796]; and cardiac and cardiovascular surgery [0.811]).

### Primary endpoints

The incidence rate of bleeding-related AEs was 18.8% (n = 398) during any type of surgery in the rTM group and 24.8% in the non-rTM group (n = 526) (**Table 2**). Overall, the incidence of total bleeding-related AEs was significantly lower (p <0.001) in the rTM group compared with that in the non-rTM group (risk ratio 0.757, 95% CI 0.674–0.849).

**Table 2:** Bleeding-related adverse events with an incidence >1% in patients undergoing any type of surgery.

Bleeding-related adverse events	Groups (n = 2117 per group)	Incidence (%)	Risk ratio		
			Point estimate	95% CI	p-value
All bleeding-related adverse events	non-rTM group	526 (24.8)	1.000	-	0.0000
	rTM group	398 (18.8)	0.757	0.674–0.849	
Intracranial hemorrhage	non-rTM group	28 (1.3)	1.000	-	0.0084
	rTM group	11 (0.5)	0.393	0.196–0.787	
Gastrointestinal hemorrhage	non-rTM group	49 (2.3)	1.000	-	0.1561
	rTM group	36 (1.7)	0.735	0.480–1.125	
Wound hemorrhage	non-rTM group	23 (1.1)	1.000	-	0.1958
	rTM group	15 (0.7)	0.652	0.341–1.246	
Other hemorrhage	non-rTM group	453 (21.4)	1.000	-	0.0000
	rTM group	335 (15.8)	0.740	0.651–0.840	
Hemorrhagic shock	non-rTM group	251 (11.9)	1.000	-	0.0000
	rTM group	150 (7.1)	0.598	0.493–0.725	
Hemorrhagic anemia	non-rTM group	140 (6.6)	1.000	-	0.1070
	rTM group	115 (5.4)	0.821	0.647–1.043	
Postoperative anemia	non-rTM group	32 (1.5)	1.000	-	0.8990
	rTM group	31 (1.5)	0.969	0.593–1.582	
Acute blood loss anemia	non-rTM group	22 (1.0)	1.000	-	0.7669
	rTM group	24 (1.1)	1.091	0.614–1.939	
Hemorrhagic trend	non-rTM group	26 (1.2)	1.000	-	0.0100
	rTM group	10 (0.5)	0.385	0.186–0.796	

rTM, recombinant thrombomodulin; CI, confidence interval

The incidence of total bleeding-related AEs was significantly lower in the rTM group compared with that in the non-rTM group for intracranial hemorrhage (p = 0.0084), other hemorrhages (p <0.001), hemorrhagic shock (p <0.001), and hemorrhagic trend (p = 0.010).

Of note, overall, the incidence of anemia caused by acute blood loss was numerically higher in the rTM group (1.1%) compared with that in the non-rTM group (1.0%).

By surgery type, the incidence rate of bleeding-related AEs for hepatic, biliary, or pancreatic surgery was 13.0% (n = 74) in the rTM group and 15.8% (n = 90) in the non-rTM group (risk ratio 0.822, 95% CI 0.618–1.093), the incidence rate for other gastrointestinal surgery (except for hepatic, biliary, or pancreatic surgery) was 31.3% (n = 192) in the rTM group and 31.9% (n = 196) in the non-rTM group (risk ratio 0.98, 95% CI 0.831–1.155); and the incidence rate for cardiac and cardiovascular surgery was 21.4% (n = 94) in the rTM group and 32.3% (n = 142) in the non-rTM group (risk ratio 0.662, 95% CI 0.529–0.829) (**S6–S8 Tables**).

The incidence rate of bleeding-related AEs was significantly lower (p = 0.0003) for cardiac and cardiovascular surgery in the rTM group compared with the non-rTM group. Among other hemorrhages, the incidence rates of hemorrhagic shock (p = 0.0049), anemia caused by acute blood loss (p = 0.0260), and acute massive hemorrhage (p = 0.0535) were significantly lower in the rTM group compared with the non-rTM group. For patients undergoing hepatic, biliary, or pancreatic surgery, the incidence rate of hemorrhagic shock was significantly lower (p = 0.0153) in the rTM group compared with the non-rTM group. There were no significant differences in the incidence rates of bleeding-related AEs among patients undergoing other gastrointestinal surgeries. Among those undergoing hepatic, biliary, or pancreatic surgery or other gastrointestinal surgeries, patients had higher incidences of anemia caused by acute blood loss in the rTM group, although these differences did not reach statistical significance.

### Secondary endpoint

A summary of all bleeding-related AEs with an incidence >1% in all patients (any type of surgery) and in those undergoing hepatic, biliary, or pancreatic surgery; gastrointestinal surgeries; and cardiac or cardiovascular surgery requiring blood transfusion or a hemostatic procedure after the day of DIC treatment is shown in **Table 3**. The incidence of all bleeding-related AEs in the rTM group (15.0% [n = 317]) was significantly lower (p = 0.0001) compared with that (19.5% [n = 412]) in the non-rTM group in all patients requiring blood transfusion or a hemostatic procedure after the day of DIC treatment. The only significant difference between the rTM and non-rTM groups regarding the incidence of bleeding-related AEs was noted among patients undergoing cardiac or cardiovascular surgery. The incidence of all bleeding-related AEs was significantly lower among patients undergoing cardiac or cardiovascular surgery treated with rTM (18.5% [n = 81]) compared with patients treated with non-rTM drugs (30.1% [n = 132]). Similarly, significant differences in favor of rTM treatment were observed for hemorrhagic shock (p = 0.0024), anemia caused by acute blood loss (p = 0.0260), and acute massive hemorrhage (p = 0.0535).

**Table 3:** Summary of bleeding-related adverse events with an incidence >1% in all patients and in those undergoing any type of surgery, hepatic, biliary, or pancreatic surgery; gastrointestinal surgeries; and cardiac or cardiovascular surgery requiring blood transfusion or a hemostatic procedure after the day of DIC treatment.

All bleeding-related adverse events	Groups	Patients (n)	Incidence (%)	Risk ratio		
				Point estimate	95% CI	p-value
All bleeding-related adverse events in patients undergoing any type of surgery	non-rTM group	2117	412 (19.5)	1.000	-	0.0001
	rTM group		317 (15.0)	0.769	0.673–0.879	
All bleeding-related adverse events in patients undergoing hepatic, biliary, or pancreatic surgery	non-rTM group	568	66 (11.6)	1.000	-	0.4470
	rTM group		58 (10.2)	0.879	0.630–1.226	
All bleeding-related adverse events in patients undergoing gastrointestinal surgeries	non-rTM group	614	156 (25.4)	1.000	-	0.6439
	rTM group		149 (24.3)	0.955	0.786–1.160	
All bleeding-related adverse events in patients undergoing cardiac or cardiovascular surgery	non-rTM group	439	132 (30.1)	1.000	-	0.0001
	rTM group		81 (18.5)	0.614	0.481–0.782	

DIC, disseminated intravascular coagulation; rTM, recombinant thrombomodulin; CI, confidence interval

Results of bleeding-related AEs with an incidence >1% in patients requiring blood transfusion or a hemostatic procedure after the day of DIC treatment in the rTM/non-rTM groups by surgery type are shown in **S9–S12 Tables**. Significant differences in favor of rTM treatment were observed for intracranial hemorrhage (p = 0.0070) and other hemorrhage (p = 0.0003) (**S9 Table**). Among other hemorrhages, significant differences in favor of rTM treatment were observed for hemorrhagic shock (p = 0.0001), hemorrhagic anemia (p = 0.0261), and hemorrhagic trend (p = 0.0198). Among all bleeding-related AEs, hemorrhagic shock had the highest incidence in both non-rTM (8.5%) and rTM groups (5.5%).

## Discussion

This study aimed to determine if there was a difference in the incidence of bleeding-related AEs between surgical patients treated with rTM and those receiving other DIC treatments. Thus, we conducted this analysis using data from a large population collected and recorded in a large medical information database. We addressed potential sources of bias in the inclusion and exclusion criteria, definitions of exposure and outcomes, and patient background variables used for the propensity score calculation by referring to previous studies when developing the study protocol [17, 18]. Additionally, the duration of the observation period included evaluations of patients 1 month after the occurrence of the bleeding-related AE. As surgery could influence the onset of bleeding-related AEs, we also investigated the incidence of these by type of surgery (surgery of the liver, gallbladder, and pancreas; surgery related to other gastrointestinal organs; and surgery related to the heart and cardiovascular system). Moreover, we investigated the incidence of bleeding-related AEs when blood transfusion or hemostatic procedure was administered to treat DIC.

In this study, we found a gap in the incidence rate of bleeding-related AEs; the incidence of bleeding-related AEs as found in this study was 15.0% in rTM groups (**Table 3**), while the estimated incidence of bleeding-related AEs according to the product information was 5.5% in the planning stage of this study. This estimated number of 5.5% was the result of a previous surveillance report under actual clinical conditions in Japan. Due to the characteristics of the MDV database, patients in this study were treated at large hospitals. Conversely, in our study, patients from small-scale hospitals were included in the data for the estimated incidence of bleeding-related AEs. Thus, it is possible that patients with a high risk of bleeding were among the patients in our study. As this was a database study and the sample size was limited beforehand, the power was calculated for the primary analyses. As a result, in the primary analysis, the estimated risk ratio was between 0.7 and 0.8 and the power was considered to be retained.

In this study, the incidence rate of total bleeding-related AEs for any surgery was significantly lower in the rTM group compared with that in the non-rTM group. Similar results were obtained for the three types of surgery evaluated (hepatic, biliary, or pancreatic surgery; other gastrointestinal surgery; and cardiac and cardiovascular surgery) requiring blood transfusion or hemostatic procedure the day after DIC treatment. Anemia caused by acute blood loss was more common in the rTM group overall and among those undergoing hepatic, biliary, or pancreatic surgery or other gastrointestinal surgeries, so special attention to this event is necessary. However, there were very few events; thus, the estimated values are not robust.

The most frequently reported bleeding events post-surgery occurred after cardiac surgery (47.4%), non-cardiac thoracic surgery (34.3%), and vascular surgery (31.5%) [19]. Our study results indicate that among all bleeding-related AEs, hemorrhagic shock occurred more frequently in both non-rTM and rTM groups. This finding is in accord with the particular impact of cardiac and cardiovascular surgery. By conducting further analyses of our data, it was possible to reveal differences in bleeding-related AEs by surgery type.

The findings of the present study support our hypothesis that rTM treatment for patients diagnosed with DIC after surgery does not increase the incidence of bleeding-related AEs compared with other DIC treatments. This is clinically relevant as it suggests that rTM treatment is a suitable and safe drug among the existing anticoagulants for DIC treatment. It is important to select the appropriate drug to treat DIC, as it is difficult to achieve sufficient anticoagulation in DIC without triggering bleeding events. Nevertheless, anticoagulation is still necessary in DIC given the state of hypercoagulability [3].

In a previous medical database study on rTM administration in patients with DIC [17], Tagami et al. compared the death rates between the rTM group and the control group in patients with severe pneumonia complicated with sepsis-related DIC and reported that there was no significant difference between groups. The study by Tagami et al., however, differed from our study in that their patients had a more severe type of DIC (i.e., sepsis-related DIC), which may explain the difference in results. Murata et al. [18] conducted an observational study comparing the in-hospital mortality, length of hospitalization, and medical costs between the rTM group and the AT-III group in inpatients with DIC associated with infectious diseases. In that study, there was no significant difference in in-hospital mortality between treatment groups; however, the use of rTM was associated with a significant decrease in the length of hospitalization and medical cost in the rTM group compared with the AT-III group. Although this study differed from ours in terms of study design, comparator, and type of DIC that patients presented, there were significant differences in terms of beneficial effects achieved by those treated with rTM compared to AT-III.

This study had several limitations. Because of the characteristics of the MDV database, we could only obtain monthly disease course data, making it difficult to assess the context of surgery during the month of DIC onset or DIC onset with the medical management administered. We were not able to collect reliable data on death as only data on death at the time of discharge were collectable. Additionally, we could only obtain some laboratory test result data (such as platelet count, creatinine, bilirubin, and FDP/D-dimer) in approximately 10% of patients, and thus, we could not consider these factors for matching using propensity scores. However, we did consider concomitant medication for complications related to DIC as a confounding factor. We could not capture information that was not included in the MDV database. For example, we could not differentiate between centers that do and those that do not have emergency departments. Thus, except for the level of care of the centers (i.e., number of beds), detailed information of the centers in which the patients were treated could not be obtained. We acknowledge that the center at which the patient was treated can influence the diagnosis of bleeding, as the examinations, surgical procedures, and indication and administration of blood transfusions, among other factors, tend to differ across centers. Finally, the results of this study cannot be generalized beyond postsurgical Japanese patients who present with DIC.

The results of this cohort study based on data from a large population obtained from the MDV database indicate that rTM treatment for Japanese postsurgical patients who develop DIC was associated with significantly less bleeding-related AEs compared with those receiving other DIC treatments. Our findings may be relevant for clinicians when selecting the most appropriate DIC treatment. In addition to addressing the limitations of the database study design, future research will focus on collecting further data to improve our understanding of the relationship between rTM treatment and bleeding-related AEs in this patient population.

## Supporting information

S1 STROBE Checklist (DOCX)

S1 Fig Flow diagram illustrating patient enrollment by hepatic, biliary, or pancreatic surgery. MDV, Medical Data Vision Co., Ltd.; rTM, recombinant thrombomodulin; DIC, disseminated intravascular coagulation; ICH, intracranial hemorrhage.
(PDF)

S2 Fig Flow diagram illustrating patient enrollment by other gastrointestinal surgery. MDV, Medical Data Vision Co., Ltd.; rTM, recombinant thrombomodulin; DIC, disseminated intravascular coagulation; ICH, intracranial hemorrhage.
(PDF)

S3 Fig Flow diagram illustrating patient enrollment by cardiac/cardiovascular surgery. MDV, Medical Data Vision Co., Ltd.; rTM, recombinant thrombomodulin; DIC, disseminated intravascular coagulation; ICH, intracranial hemorrhage.
(PDF)

S1 Table List of bleeding-related adverse events. (DOCX)

S2 Table Usage ratio of the most frequently used drugs in the non-rTM treatment group. (DOCX)

S3 Table Demographic and clinical baseline characteristics of patients who underwent hepatic, biliary, or pancreatic surgery. ^a^Includes diseases that seem to be severe (e.g., liver cirrhosis), but as defined in the previous study (Quan et al. Med Care. 2005;43:1130–9.)
^b^When the test was performed a few times on the same day, we adopted the average.
^c^When the test was performed a few times on the same day, we adopted the minimum (sensitivity analysis).
^d^When the test was performed a few times on the same day, we adopted the maximum (sensitivity analysis).
rTM, recombinant thrombomodulin; DIC, disseminated intravascular coagulation; FDP, fibrinogen/fibrin degradation products.
(DOCX)

S4 Table Demographic and clinical baseline characteristics of patients who underwent other gastrointestinal surgery. ^a^Includes diseases that seem to be severe (e.g., liver cirrhosis), but as defined in the previous study (Quan et al. Med Care. 2005;43:1130–9.)
^b^When the test was performed a few times on the same day, we adopted the average.
^c^When the test was performed a few times on the same day, we adopted the minimum (sensitivity analysis).
^d^When the test was performed a few times on the same day, we adopted the maximum (sensitivity analysis).
rTM, recombinant thrombomodulin; DIC, disseminated intravascular coagulation; FDP, fibrinogen/fibrin degradation products.
(DOCX)

S5 Table Demographic and clinical baseline characteristics of patients who underwent cardiac/cardiovascular surgery. ^a^Includes diseases that seem to be severe (e.g., liver cirrhosis), but as defined in the previous study (Quan et al. Med Care. 2005;43:1130–9.)
^b^When the test was performed a few times on the same day, we adopted the average.
^c^When the test was performed a few times on the same day, we adopted the minimum (sensitivity analysis).^d^When the test was performed a few times on the same day, we adopted the maximum (sensitivity analysis).
rTM, recombinant thrombomodulin; DIC, disseminated intravascular coagulation; FDP, fibrinogen/fibrin degradation products.
(DOCX)

S6 Table Bleeding-related adverse events with an incidence >1% in patients undergoing hepatic, biliary, or pancreatic surgery. rTM, recombinant thrombomodulin; CI, confidence interval.
(DOCX)

S7 Table Bleeding-related adverse events with an incidence >1% in patients undergoing other gastrointestinal surgeries. rTM, recombinant thrombomodulin; CI, confidence interval.
(DOCX)

S8 Table Bleeding-related adverse events with an incidence >1% in patients undergoing cardiac or cardiovascular surgery. rTM, recombinant thrombomodulin; CI, confidence interval.
(DOCX)

S9 Table Bleeding-related adverse events with an incidence >1% in patients undergoing any type of surgery requiring blood transfusion or a hemostatic procedure after the day of DIC treatment DIC, disseminated intravascular coagulation; rTM, recombinant thrombomodulin; CI, confidence interval. (DOCX)

S10 Table Bleeding-related adverse events with an incidence >1% in patients undergoing hepatic, biliary, or pancreatic surgery requiring blood transfusion or a hemostatic procedure after the day of DIC treatment. DIC, disseminated intravascular coagulation; rTM, recombinant thrombomodulin; CI, confidence interval.
(DOCX)

S11 Table Bleeding-related adverse events with an incidence >1% in patients undergoing gastrointestinal surgeries requiring blood transfusion or a hemostatic procedure after the day of DIC treatment. DIC, disseminated intravascular coagulation; rTM, recombinant thrombomodulin; CI, confidence interval.
(DOCX)

S12 Table Bleeding-related adverse events with an incidence >1% in patients undergoing cardiac or cardiovascular surgery requiring blood transfusion or a hemostatic procedure after the day of DIC treatment. DIC, disseminated intravascular coagulation; rTM, recombinant thrombomodulin; CI, confidence interval.
(DOCX)
