# Supplementary material for: Assessment of bleeding in patients with disseminated intravascular coagulation after receiving surgery and recombinant human soluble thrombomodulin: A cohort study using a database
Source: PLoS One. 2018 Oct 8;13(10):e0205146. doi: 10.1371/journal.pone.0205146 (PMC6175500; doi:10.1371/journal.pone.0205146)
Supplement: S1 STROBE Checklist — (DOCX) [file pone.0205146.s001.docx]

STROBE Statement—checklist of items that should be included in reports of observational studies

|  | Item No. | Recommendation | Page  No. | Relevant text from manuscript |
| --- | --- | --- | --- | --- |
| **Title and abstract** | 1 | (*a*) Indicate the study’s design with a commonly used term in the title or the abstract | Page 1 / page 2 | Assessment of bleeding in patients with disseminated intravascular coagulation after receiving surgery and recombinant human soluble thrombomodulin: A cohort study using a database /In this cohort study, data were obtained from a large medical database (22 centers in Japan). |
|  |  | (*b*) Provide in the abstract an informative and balanced summary of what was done and what was found | Page 2 | Our findings suggest that …with those receiving other DIC treatments. |
| Introduction | | | |  |
| Background/rationale | 2 | Explain the scientific background and rationale for the investigation being reported | Page 3 | A Japanese study based on a national administrative database reported that… within 28 days was 27.7% in 2012 [2]. |
| Objectives | 3 | State specific objectives, including any prespecified hypotheses | Page 4 | For the present study, we hypothesized that… and the group receiving other DIC treatments. |
| Methods | | | |  |
| Study design | 4 | Present key elements of study design early in the paper | Page 5 | This was a cohort study using data from 22 centers in Japan obtained from a large medical database collected by MDV. |
| Setting | 5 | Describe the setting, locations, and relevant dates, including periods of recruitment, exposure, follow-up, and data collection | Page 5 and 6 | All participants were Japanese. Participants were divided into two groups…and 31 August 2015. |
| Participants | 6 | (*a*) *Cohort study*—Give the eligibility criteria, and the sources and methods of selection of participants. Describe methods of follow-up  *Case-control study*—Give the eligibility criteria, and the sources and methods of case ascertainment and control selection. Give the rationale for the choice of cases and controls  *Cross-sectional study*—Give the eligibility criteria, and the sources and methods of selection of participants | Page 6 and 7 | The target population of the present study included patients who attended emergency rooms… but no initial prescription of rTM on the day of the surgery or within 1 week after surgery. |
|  |  | (*b*) *Cohort study*—For matched studies, give matching criteria and number of exposed and unexposed  *Case-control study*—For matched studies, give matching criteria and the number of controls per case | Page 9 | The present analysis was performed on selected patients from the database… (the propensity score at which both the rTM and non-rTM groups coexist) were excluded from the analysis. |
| Variables | 7 | Clearly define all outcomes, exposures, predictors, potential confounders, and effect modifiers. Give diagnostic criteria, if applicable | Page 7 and 8 | Measurements and variables… Additionally, the duration of the observation period included evaluations of patients 1 month after the occurrence of the bleeding-related AE. |
| Data sources/ measurement | 8* | For each variable of interest, give sources of data and details of methods of assessment (measurement). Describe comparability of assessment methods if there is more than one group | Page 5 / page 7 | This was a cohort study using data from 22 centers in Japan obtained from… approximately 18,630,000 pediatric and adult patients have been registered in the database. /  Primary endpoint… not blood transfusion or a hemostatic procedure was administered after the day of DIC treatment. |
| Bias | 9 | Describe any efforts to address potential sources of bias | Page 29 | We addressed potential sources of bias… 1 month after the occurrence of the bleeding-related AE. |
| Study size | 10 | Explain how the study size was arrived at | Page 8 | Study size… for 2150 patients per group. |

Continued on next page

| Quantitative variables | 11 | Explain how quantitative variables were handled in the analyses. If applicable, describe which groupings were chosen and why | Page 8 | Bias... 1 month after the occurrence of the bleeding-related AE. |
| --- | --- | --- | --- | --- |
| Statistical methods | 12 | (*a*) Describe all statistical methods, including those used to control for confounding | Page 9 | Patient background characteristics are presented as n (%)…SAS v 9.4 (SAS Institute, Cary, NC, USA) was used for conducting the statistical analyses. |
|  |  | (*b*) Describe any methods used to examine subgroups and interactions | NA |  |
|  |  | (*c*) Explain how missing data were addressed | NA |  |
|  |  | (*d*) *Cohort study*—If applicable, explain how loss to follow-up was addressed  *Case-control study*—If applicable, explain how matching of cases and controls was addressed  *Cross-sectional study*—If applicable, describe analytical methods taking account of sampling strategy | NA |  |
|  |  | (*e*) Describe any sensitivity analyses | NA |  |
| Results | | | | |
| Participants | 13* | (a) Report numbers of individuals at each stage of study—eg numbers potentially eligible, examined for eligibility, confirmed eligible, included in the study, completing follow-up, and analysed | Page 9 and 10 | Fig 1 shows the patient disposition and the number of patients… These were matched by age and sex. |
|  |  | (b) Give reasons for non-participation at each stage |  | Fig 1 |
|  |  | (c) Consider use of a flow diagram |  | Fig 1 |
| Descriptive data | 14* | (a) Give characteristics of study participants (eg demographic, clinical, social) and information on exposures and potential confounders |  | Table 1 |
|  |  | (b) Indicate number of participants with missing data for each variable of interest | NA |  |
|  |  | (c) *Cohort study*—Summarise follow-up time (eg, average and total amount) | NA |  |
| Outcome data | 15* | *Cohort study*—Report numbers of outcome events or summary measures over time | Page24-30 |  |
|  |  | *Case-control study—*Report numbers in each exposure category, or summary measures of exposure | NA |  |
|  |  | *Cross-sectional study—*Report numbers of outcome events or summary measures | NA |  |
| Main results | 16 | (*a*) Give unadjusted estimates and, if applicable, confounder-adjusted estimates and their precision (eg, 95% confidence interval). Make clear which confounders were adjusted for and why they were included | Page 24- page 27 |  |
|  |  | (*b*) Report category boundaries when continuous variables were categorized | NA |  |
|  |  | (*c*) If relevant, consider translating estimates of relative risk into absolute risk for a meaningful time period | NA |  |

Continued on next page

| Other analyses | 17 | Report other analyses done—eg analyses of subgroups and interactions, and sensitivity analyses | NA |  |
| --- | --- | --- | --- | --- |
| Discussion | | | | |
| Key results | 18 | Summarise key results with reference to study objectives | Page 27 | This study aimed to determine if there was a difference in the incidence of bleeding-related AEs… was still significantly lower in the rTM group compared with that in the non-rTM group. |
| Limitations | 19 | Discuss limitations of the study, taking into account sources of potential bias or imprecision. Discuss both direction and magnitude of any potential bias | Page 29 | This study had several limitations. B… The results of this study cannot be generalized beyond postsurgical Japanese patients who present with DIC. |
| Interpretation | 20 | Give a cautious overall interpretation of results considering objectives, limitations, multiplicity of analyses, results from similar studies, and other relevant evidence | Page 28 and page 29 | In a previous medical database study on rTM administration in patients with DIC [17]…in terms of beneficial effects achieved by those treated with rTM compared to AT-III. |
| Generalisability | 21 | Discuss the generalisability (external validity) of the study results | Page 29 | The results of this cohort study based on data from a large population… relationship between rTM treatment and bleeding-related AEs in this patient population. |
| Other information | |  | | |
| Funding | 22 | Give the source of funding and the role of the funders for the present study and, if applicable, for the original study on which the present article is based | Page 33 | This study was funded by Asahi Kasei Pharma Corporation… was funded by Asahi Kasei Pharma Corporation |

*Give information separately for cases and controls in case-control studies and, if applicable, for exposed and unexposed groups in cohort and cross-sectional studies.

**Note:** An Explanation and Elaboration article discusses each checklist item and gives methodological background and published examples of transparent reporting. The STROBE checklist is best used in conjunction with this article (freely available on the Web sites of PLoS Medicine at http://www.plosmedicine.org/, Annals of Internal Medicine at http://www.annals.org/, and Epidemiology at http://www.epidem.com/). Information on the STROBE Initiative is available at www.strobe-statement.org.
