# Supplementary material for: Assessment of bleeding in patients with disseminated intravascular coagulation after receiving surgery and recombinant human soluble thrombomodulin: A cohort study using a database
Source: PLoS One. 2018 Oct 8;13(10):e0205146. doi: 10.1371/journal.pone.0205146 (PMC6175500; doi:10.1371/journal.pone.0205146)

S1 Fig Flow diagram illustrating patient enrollment by hepatic, biliary, or pancreatic surgery

Abbreviations:
MDV, Medical Data Vision Co., Ltd.;
rTM, recombinant thrombomodulin;
DIC, disseminated intravascular coagulation;
ICH, intracranial hemorrhage

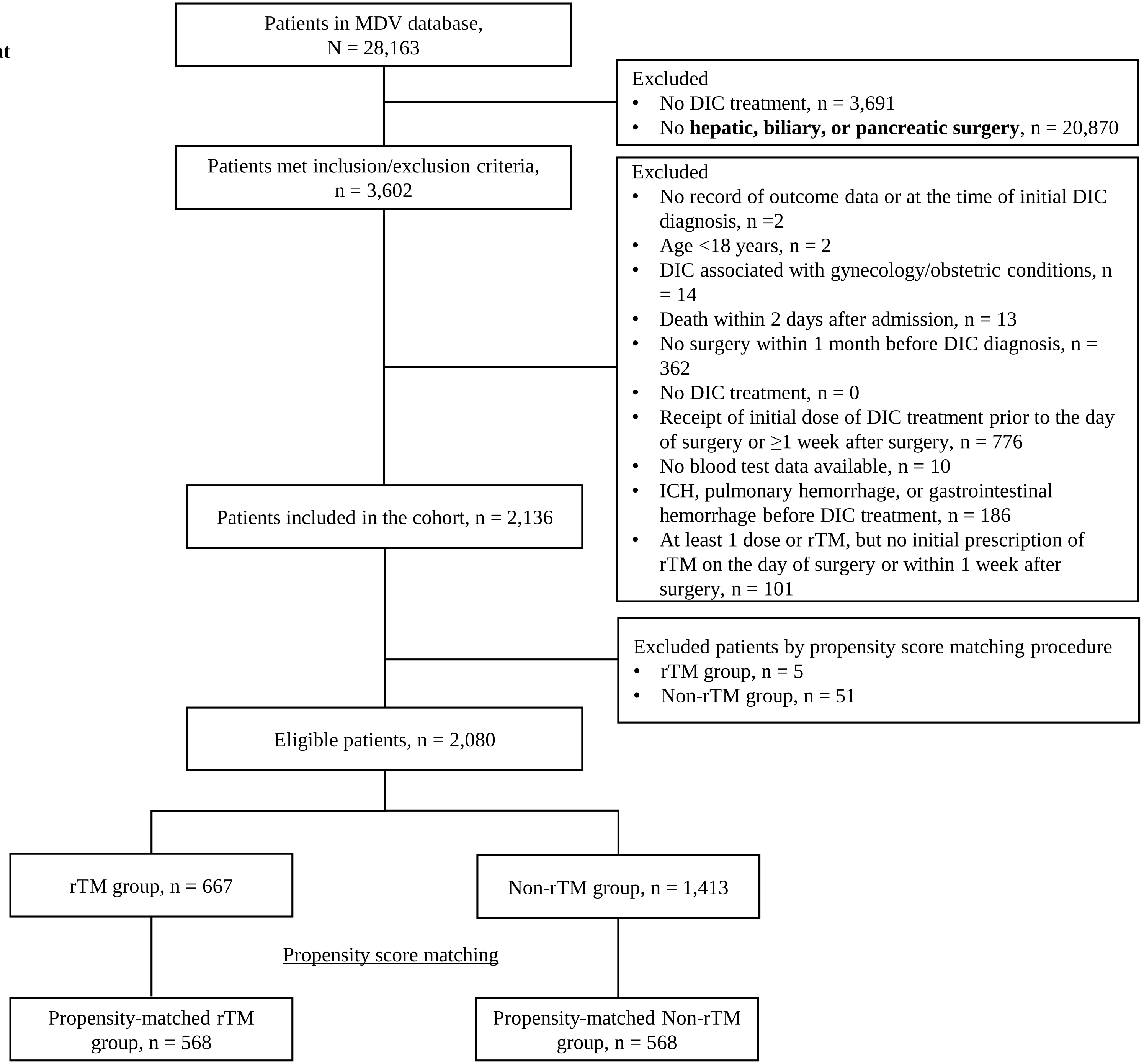

Supplement: S1 Fig — MDV, Medical Data Vision Co., Ltd.; rTM, recombinant thrombomodulin; DIC, disseminated intravascular coagulation; ICH, intracranial hemorrhage. (PDF) [file pone.0205146.s002.pdf]
