# Supplementary material for: Assessment of bleeding in patients with disseminated intravascular coagulation after receiving surgery and recombinant human soluble thrombomodulin: A cohort study using a database
Source: PLoS One. 2018 Oct 8;13(10):e0205146. doi: 10.1371/journal.pone.0205146 (PMC6175500; doi:10.1371/journal.pone.0205146)
Supplement: S1 Table — (DOCX) [file pone.0205146.s005.docx]

**S1 Table. List of bleeding-related adverse events**

| ICD-10 code | ICD-10 disease name | Disease code |
| --- | --- | --- |
| A09 | Other gastroenteritis and colitis of infectious and unspecified origin | 5789014 |
| A09 | Other gastroenteritis and colitis of infectious and unspecified origin | 5789018 |
| D500 | Iron deficiency anaemia secondary to blood loss (chronic) | 2809005 |
| D62 | Acute posthaemorrhagic anaemia | 8832363 |
| D62 | Acute posthaemorrhagic anaemia | 8835357 |
| D690 | Allergic purpura | 2870012 |
| D690 | Allergic purpura | 2878004 |
| D690 | Allergic purpura | 8830390 |
| D690 | Allergic purpura | 8834160 |
| D690 | Allergic purpura | 8834703 |
| D690 | Allergic purpura | 8842546 |
| D690 | Allergic purpura | 8848314 |
| D690 | Allergic purpura | 8848315 |
| D692 | Other nonthrombocytopenic purpura | 2870019 |
| D692 | Other nonthrombocytopenic purpura | 2870026 |
| D692 | Other nonthrombocytopenic purpura | 2872010 |
| D692 | Other nonthrombocytopenic purpura | 8836522 |
| D692 | Other nonthrombocytopenic purpura | 8837961 |
| D692 | Other nonthrombocytopenic purpura | 8842636 |
| D693 | Idiopathic thrombocytopenic purpura | 2873013 |
| D693 | Idiopathic thrombocytopenic purpura | 8830887 |
| D693 | Idiopathic thrombocytopenic purpura | 8846110 |
| D693 | Idiopathic thrombocytopenic purpura | 8846222 |
| D694 | Other primary thrombocytopenia | 2280083 |
| D694 | Other primary thrombocytopenia | 2873007 |
| D694 | Other primary thrombocytopenia | 8830570 |
| D694 | Other primary thrombocytopenia | 8833302 |
| D694 | Other primary thrombocytopenia | 8833812 |
| D695 | Secondary thrombocytopenia | 2872008 |
| D695 | Secondary thrombocytopenia | 2873019 |
| D695 | Secondary thrombocytopenia | 2874005 |
| D695 | Secondary thrombocytopenia | 8836825 |
| D695 | Secondary thrombocytopenia | 8844605 |
| D698 | Other specified haemorrhagic conditions | 2878003 |
| D699 | Haemorrhagic condition, unspecified | 2869010 |
| D699 | Haemorrhagic condition, unspecified | 2872016 |
| D891 | Cryoglobulinaemia | 2732008 |
| D891 | Cryoglobulinaemia | 2732013 |
| D891 | Cryoglobulinaemia | 8832828 |
| D891 | Cryoglobulinaemia | 8832829 |
| D891 | Cryoglobulinaemia | 8832830 |
| D891 | Cryoglobulinaemia | 8832831 |
| D891 | Cryoglobulinaemia | 8832832 |
| D891 | Cryoglobulinaemia | 8833301 |
| D891 | Cryoglobulinaemia | 8836823 |
| D891 | Cryoglobulinaemia | 8840105 |
| G361 | Acute and subacute haemorrhagic leukoencephalitis [Hurst] | 8832371 |
| G951 | Vascular myelopathies | 3361005 |
| G951 | Vascular myelopathies | 3361013 |
| G951 | Vascular myelopathies | 3361019 |
| G951 | Vascular myelopathies | 4320004 |
| G968 | Other specified disorders of central nervous system | 8835981 |
| H113 | Conjunctival haemorrhage | 3727006 |
| H113 | Conjunctival haemorrhage | 8831968 |
| H168 | Other keratitis | 8834634 |
| H208 | Other iridocyclitis | 8834639 |
| H210 | Hyphaema | 3644003 |
| H210 | Hyphaema | 8836580 |
| H313 | Choroidal haemorrhage and rupture | 8840453 |
| H356 | Retinal haemorrhage | 3628002 |
| H356 | Retinal haemorrhage | 3628013 |
| H356 | Retinal haemorrhage | 3628018 |
| H356 | Retinal haemorrhage | 3628019 |
| H356 | Retinal haemorrhage | 3628022 |
| H356 | Retinal haemorrhage | 3628025 |
| H356 | Retinal haemorrhage | 8830975 |
| H356 | Retinal haemorrhage | 8834734 |
| H356 | Retinal haemorrhage | 8840624 |
| H356 | Retinal haemorrhage | 8840631 |
| H356 | Retinal haemorrhage | 8840635 |
| H431 | Vitreous haemorrhage | 3792006 |
| H431 | Vitreous haemorrhage | 8842347 |
| H470 | Disorders of optic nerve, not elsewhere classified | 8834333 |
| H470 | Disorders of optic nerve, not elsewhere classified | 8834344 |
| H603 | Other infective otitis externa | 8834635 |
| H669 | Otitis media, unspecified | 8834642 |
| H738 | Other specified disorders of tympanic membrane | 3848003 |
| H922 | Otorrhagia | 3809001 |
| H922 | Otorrhagia | 3888004 |
| I312 | Haemopericardium, not elsewhere classified | 8835136 |
| I312 | Haemopericardium, not elsewhere classified | 8835137 |
| I600 | Subarachnoid haemorrhage from carotid siphon and bifurcation | 8838299 |
| I600 | Subarachnoid haemorrhage from carotid siphon and bifurcation | 8846321 |
| I600 | Subarachnoid haemorrhage from carotid siphon and bifurcation | 8847449 |
| I600 | Subarachnoid haemorrhage from carotid siphon and bifurcation | 8847541 |
| I601 | Subarachnoid haemorrhage from middle cerebral artery | 8837619 |
| I601 | Subarachnoid haemorrhage from middle cerebral artery | 8847527 |
| I602 | Subarachnoid haemorrhage from anterior communicating artery | 8836504 |
| I602 | Subarachnoid haemorrhage from anterior communicating artery | 8847505 |
| I603 | Subarachnoid haemorrhage from posterior communicating artery | 8833433 |
| I603 | Subarachnoid haemorrhage from posterior communicating artery | 8847468 |
| I604 | Subarachnoid haemorrhage from basilar artery | 8838740 |
| I604 | Subarachnoid haemorrhage from basilar artery | 8847545 |
| I605 | Subarachnoid haemorrhage from vertebral artery | 8837825 |
| I605 | Subarachnoid haemorrhage from vertebral artery | 8847531 |
| I606 | Subarachnoid haemorrhage from other intracranial arteries | 8846593 |
| I606 | Subarachnoid haemorrhage from other intracranial arteries | 8846750 |
| I606 | Subarachnoid haemorrhage from other intracranial arteries | 8847469 |
| I606 | Subarachnoid haemorrhage from other intracranial arteries | 8847506 |
| I607 | Subarachnoid haemorrhage from intracranial artery, unspecified | 8847536 |
| I608 | Other subarachnoid haemorrhage | 8835797 |
| I608 | Other subarachnoid haemorrhage | 8847896 |
| I609 | Subarachnoid haemorrhage, unspecified | 4309001 |
| I609 | Subarachnoid haemorrhage, unspecified | 4309005 |
| I609 | Subarachnoid haemorrhage, unspecified | 8836339 |
| I609 | Subarachnoid haemorrhage, unspecified | 8838751 |
| I610 | Intracerebral haemorrhage in hemisphere, subcortical | 4310038 |
| I610 | Intracerebral haemorrhage in hemisphere, subcortical | 4319027 |
| I610 | Intracerebral haemorrhage in hemisphere, subcortical | 4319030 |
| I610 | Intracerebral haemorrhage in hemisphere, subcortical | 8847680 |
| I611 | Intracerebral haemorrhage in hemisphere, cortical | 8839257 |
| I613 | Intracerebral haemorrhage in brain stem | 4319013 |
| I613 | Intracerebral haemorrhage in brain stem | 8841358 |
| I613 | Intracerebral haemorrhage in brain stem | 8845147 |
| I614 | Intracerebral haemorrhage in cerebellum | 4319006 |
| I615 | Intracerebral haemorrhage, intraventricular | 4319018 |
| I615 | Intracerebral haemorrhage, intraventricular | 4319032 |
| I616 | Intracerebral haemorrhage, multiple localized | 8836998 |
| I618 | Other intracerebral haemorrhage | 4320007 |
| I619 | Intracerebral haemorrhage, unspecified | 4319003 |
| I619 | Intracerebral haemorrhage, unspecified | 4319009 |
| I619 | Intracerebral haemorrhage, unspecified | 4319020 |
| I619 | Intracerebral haemorrhage, unspecified | 8847897 |
| I620 | Subdural haemorrhage (acute)(nontraumatic) | 4321006 |
| I620 | Subdural haemorrhage (acute)(nontraumatic) | 4321008 |
| I620 | Subdural haemorrhage (acute)(nontraumatic) | 8833690 |
| I620 | Subdural haemorrhage (acute)(nontraumatic) | 8843499 |
| I621 | Nontraumatic extradural haemorrhage | 8843500 |
| I629 | Intracranial haemorrhage (nontraumatic), unspecified | 8839202 |
| I638 | Other cerebral infarction | 4341044 |
| I711 | Thoracic aortic aneurysm, ruptured | 4411002 |
| I711 | Thoracic aortic aneurysm, ruptured | 8838989 |
| I713 | Abdominal aortic aneurysm, ruptured | 4413002 |
| I713 | Abdominal aortic aneurysm, ruptured | 8838991 |
| I715 | Thoracoabdominal aortic aneurysm, ruptured | 8832571 |
| I715 | Thoracoabdominal aortic aneurysm, ruptured | 8838988 |
| I788 | Other diseases of capillaries | 4489012 |
| I850 | Oesophageal varices with bleeding | 4560002 |
| I864 | Gastric varices | 8845850 |
| J90 | Pleural effusion, not elsewhere classified | 8833110 |
| J942 | Haemothorax | 5118002 |
| J942 | Haemothorax | 5118003 |
| J950 | Tracheostomy malfunction | 8832181 |
| K068 | Other specified disorders of gingiva and edentulous alveolar ridge | 8834466 |
| K092 | Other cysts of jaw | 8834637 |
| K121 | Other forms of stomatitis | 5280051 |
| K137 | Other and unspecified lesions of oral mucosa | 5289010 |
| K148 | Other diseases of tongue | 8836434 |
| K226 | Gastro-oesophageal laceration-haemorrhage syndrome | 8840294 |
| K228 | Other specified diseases of oesophagus | 5308005 |
| K250 | Gastric ulcer （Acute with haemorrhage） | 5319011 |
| K254 | Gastric ulcer （Chronic or unspecified with haemorrhage） | 8834632 |
| K254 | Gastric ulcer （Chronic or unspecified with haemorrhage） | 8847788 |
| K260 | Duodenal ulcer (Acute with haemorrhage) | 8845123 |
| K264 | Duodenal ulcer (Chronic or unspecified with haemorrhage) | 8834641 |
| K284 | Gastrojejunal ulcer (Chronic or unspecified with haemorrhage) | 8847762 |
| K290 | Acute haemorrhagic gastritis | 8834631 |
| K571 | Diverticular disease of small intestine without perforation or abscess | 8845800 |
| K573 | Diverticular disease of large intestine without perforation or abscess | 8845742 |
| K573 | Diverticular disease of large intestine without perforation or abscess | 8845749 |
| K573 | Diverticular disease of large intestine without perforation or abscess | 8845763 |
| K573 | Diverticular disease of large intestine without perforation or abscess | 8845806 |
| K573 | Diverticular disease of large intestine without perforation or abscess | 8845814 |
| K625 | Haemorrhage of anus and rectum | 5693001 |
| K625 | Haemorrhage of anus and rectum | 5789011 |
| K625 | Haemorrhage of anus and rectum | 8833703 |
| K659 | Peritonitis, unspecified | 5672027 |
| K661 | Haemoperitoneum | 8839651 |
| K661 | Haemoperitoneum | 8839763 |
| K661 | Haemoperitoneum | 8841643 |
| K768 | Other specified diseases of liver | 8831551 |
| K85 | Acute pancreatitis | 8832368 |
| K85 | Acute pancreatitis | 8832370 |
| K920 | Haematemesis | 5780005 |
| K921 | Melaena | 5781001 |
| K921 | Melaena | 5781002 |
| K921 | Melaena | 5789005 |
| K921 | Melaena | 7921002 |
| K922 | Gastrointestinal haemorrhage, unspecified | 5780004 |
| K922 | Gastrointestinal haemorrhage, unspecified | 5789001 |
| K922 | Gastrointestinal haemorrhage, unspecified | 5789007 |
| K922 | Gastrointestinal haemorrhage, unspecified | 5789008 |
| K922 | Gastrointestinal haemorrhage, unspecified | 8837732 |
| K922 | Gastrointestinal haemorrhage, unspecified | 8848141 |
| L270 | Generalized skin eruption due to drugs and medicaments | 8834475 |
| M250 | Haemarthrosis | 8831595 |
| M250 | Haemarthrosis | 9241024 |
| M303 | Mucocutaneous lymph node syndrome [Kawasaki] | 4461004 |
| M311 | Thrombotic microangiopathy | 4466002 |
| M311 | Thrombotic microangiopathy | 8847881 |
| N029 | Recurrent and persistent haematuria (Unspecified) | 5997004 |
| N029 | Recurrent and persistent haematuria (Unspecified) | 5997005 |
| N029 | Recurrent and persistent haematuria (Unspecified) | 5997012 |
| N029 | Recurrent and persistent haematuria (Unspecified) | 5997016 |
| N029 | Recurrent and persistent haematuria (Unspecified) | 8833171 |
| N029 | Recurrent and persistent haematuria (Unspecified) | 8834239 |
| N029 | Recurrent and persistent haematuria (Unspecified) | 8841019 |
| N029 | Recurrent and persistent haematuria (Unspecified) | 8841023 |
| N288 | Other specified disorders of kidney and ureter | 5938021 |
| N288 | Other specified disorders of kidney and ureter | 5938028 |
| N288 | Other specified disorders of kidney and ureter | 8835604 |
| N300 | Acute cystitis | 5950002 |
| N309 | Cystitis, unspecified | 8842024 |
| N328 | Other specified disorders of bladder | 5967004 |
| N368 | Other specified disorders of urethra | 5997011 |
| N421 | Congestion and haemorrhage of prostate | 6021002 |
| N488 | Other specified disorders of penis | 6078036 |
| N501 | Vascular disorders of male genital organs | 8830697 |
| N645 | Other signs and symptoms in breast | 6117024 |
| N645 | Other signs and symptoms in breast | 6117032 |
| N830 | Follicular cyst of ovary | 6201005 |
| N830 | Follicular cyst of ovary | 8834645 |
| N831 | Corpus luteum cyst | 8834633 |
| N838 | Other noninflammatory disorders of ovary, fallopian tube and broad ligament | 6201004 |
| N908 | Other specified noninflammatory disorders of vulva and perineum | 6245002 |
| N921 | Excessive and frequent menstruation with irregular cycle | 6268001 |
| N921 | Excessive and frequent menstruation with irregular cycle | 8834262 |
| N923 | Ovulation bleeding | 6265001 |
| N923 | Ovulation bleeding | 6265002 |
| N924 | Excessive bleeding in the premenopausal period | 6271002 |
| N930 | Postcoital and contact bleeding | 8835843 |
| N938 | Other specified abnormal uterine and vaginal bleeding | 6263004 |
| N938 | Other specified abnormal uterine and vaginal bleeding | 8832247 |
| N939 | Abnormal uterine and vaginal bleeding, unspecified | 6268005 |
| N939 | Abnormal uterine and vaginal bleeding, unspecified | 6269007 |
| N950 | Postmenopausal bleeding | 8839932 |
| N988 | Other complications associated with artificial fertilization | 8848529 |
| R040 | Epistaxis | 7847002 |
| R040 | Epistaxis | 7847003 |
| R040 | Epistaxis | 7847004 |
| R040 | Epistaxis | 7847006 |
| R040 | Epistaxis | 8839467 |
| R041 | Haemorrhage from throat | 7848002 |
| R041 | Haemorrhage from throat | 7848003 |
| R041 | Haemorrhage from throat | 7848005 |
| R041 | Haemorrhage from throat | 7848006 |
| R041 | Haemorrhage from throat | 8830641 |
| R042 | Haemoptysis | 7863003 |
| R042 | Haemoptysis | 7864002 |
| R048 | Haemorrhage from other sites in respiratory passages | 4462004 |
| R048 | Haemorrhage from other sites in respiratory passages | 4789006 |
| R048 | Haemorrhage from other sites in respiratory passages | 5191010 |
| R048 | Haemorrhage from other sites in respiratory passages | 7863001 |
| R048 | Haemorrhage from other sites in respiratory passages | 8838831 |
| R049 | Haemorrhage from respiratory passages, unspecified | 8832240 |
| R18 | Ascites | 7895003 |
| R233 | Spontaneous ecchymoses | 2879002 |
| R233 | Spontaneous ecchymoses | 7827001 |
| R233 | Spontaneous ecchymoses | 7827002 |
| R233 | Spontaneous ecchymoses | 7827005 |
| R233 | Spontaneous ecchymoses | 7827006 |
| R233 | Spontaneous ecchymoses | 7827007 |
| R233 | Spontaneous ecchymoses | 7827008 |
| R233 | Spontaneous ecchymoses | 7827013 |
| R233 | Spontaneous ecchymoses | 8838196 |
| R31 | Unspecified haematuria | 5997003 |
| R31 | Unspecified haematuria | 5997009 |
| R31 | Unspecified haematuria | 5997015 |
| R31 | Unspecified haematuria | 5997021 |
| R31 | Unspecified haematuria | 6260001 |
| R31 | Unspecified haematuria | 6266004 |
| R31 | Unspecified haematuria | 6269004 |
| R31 | Unspecified haematuria | 8830391 |
| R31 | Unspecified haematuria | 8833170 |
| R31 | Unspecified haematuria | 8835589 |
| R571 | Hypovolaemic shock | 9584004 |
| R58 | Haemorrhage, not elsewhere classified | 4590002 |
| R58 | Haemorrhage, not elsewhere classified | 4590003 |
| R58 | Haemorrhage, not elsewhere classified | 4590005 |
| R58 | Haemorrhage, not elsewhere classified | 4590006 |
| R58 | Haemorrhage, not elsewhere classified | 8832405 |
| R58 | Haemorrhage, not elsewhere classified | 8832664 |
| R58 | Haemorrhage, not elsewhere classified | 8834774 |
| R58 | Haemorrhage, not elsewhere classified | 8835227 |
| R58 | Haemorrhage, not elsewhere classified | 8837085 |
| S000 | Superficial injury of scalp | 9209017 |
| S000 | Superficial injury of scalp | 9209044 |
| S000 | Superficial injury of scalp | 9209049 |
| S000 | Superficial injury of scalp | 9209051 |
| S000 | Superficial injury of scalp | 9209053 |
| S000 | Superficial injury of scalp | 9209056 |
| S000 | Superficial injury of scalp | 9209057 |
| S000 | Superficial injury of scalp | 9209059 |
| S000 | Superficial injury of scalp | 9209061 |
| S000 | Superficial injury of scalp | 9209062 |
| S001 | Contusion of eyelid and periocular area | 8831973 |
| S001 | Contusion of eyelid and periocular area | 8831989 |
| S001 | Contusion of eyelid and periocular area | 8831990 |
| S001 | Contusion of eyelid and periocular area | 8832021 |
| S001 | Contusion of eyelid and periocular area | 8832022 |
| S001 | Contusion of eyelid and periocular area | 8832058 |
| S001 | Contusion of eyelid and periocular area | 8832067 |
| S001 | Contusion of eyelid and periocular area | 8832068 |
| S001 | Contusion of eyelid and periocular area | 8836477 |
| S001 | Contusion of eyelid and periocular area | 8836485 |
| S001 | Contusion of eyelid and periocular area | 9209042 |
| S001 | Contusion of eyelid and periocular area | 9211001 |
| S003 | Superficial injury of nose | 8839530 |
| S003 | Superficial injury of nose | 8839531 |
| S003 | Superficial injury of nose | 9209071 |
| S004 | Superficial injury of ear | 8831801 |
| S004 | Superficial injury of ear | 8831809 |
| S004 | Superficial injury of ear | 8831810 |
| S004 | Superficial injury of ear | 8835162 |
| S004 | Superficial injury of ear | 8835173 |
| S004 | Superficial injury of ear | 8835174 |
| S005 | Superficial injury of lip and oral cavity | 8833396 |
| S005 | Superficial injury of lip and oral cavity | 8833410 |
| S005 | Superficial injury of lip and oral cavity | 8833471 |
| S005 | Superficial injury of lip and oral cavity | 8833487 |
| S005 | Superficial injury of lip and oral cavity | 8833488 |
| S005 | Superficial injury of lip and oral cavity | 8834472 |
| S005 | Superficial injury of lip and oral cavity | 8835413 |
| S005 | Superficial injury of lip and oral cavity | 9209020 |
| S005 | Superficial injury of lip and oral cavity | 9209063 |
| S007 | Multiple superficial injuries of head | 8832115 |
| S007 | Multiple superficial injuries of head | 8832116 |
| S007 | Multiple superficial injuries of head | 8838127 |
| S007 | Multiple superficial injuries of head | 8838135 |
| S007 | Multiple superficial injuries of head | 9209013 |
| S008 | Superficial injury of other parts of head | 8831119 |
| S008 | Superficial injury of other parts of head | 8831136 |
| S008 | Superficial injury of other parts of head | 8831865 |
| S008 | Superficial injury of other parts of head | 8832124 |
| S008 | Superficial injury of other parts of head | 8832593 |
| S008 | Superficial injury of other parts of head | 8832622 |
| S008 | Superficial injury of other parts of head | 8832996 |
| S008 | Superficial injury of other parts of head | 8835387 |
| S008 | Superficial injury of other parts of head | 8835402 |
| S008 | Superficial injury of other parts of head | 9209001 |
| S008 | Superficial injury of other parts of head | 9209008 |
| S008 | Superficial injury of other parts of head | 9209012 |
| S051 | Contusion of eyeball and orbital tissues | 8831172 |
| S062 | Diffuse brain injury | 8510002 |
| S062 | Diffuse brain injury | 8843472 |
| S062 | Diffuse brain injury | 8843473 |
| S062 | Diffuse brain injury | 8843485 |
| S062 | Diffuse brain injury | 8843593 |
| S063 | Focal brain injury | 8510012 |
| S063 | Focal brain injury | 8832667 |
| S063 | Focal brain injury | 8838687 |
| S063 | Focal brain injury | 8843170 |
| S063 | Focal brain injury | 8843305 |
| S063 | Focal brain injury | 8843306 |
| S063 | Focal brain injury | 8843356 |
| S063 | Focal brain injury | 8843470 |
| S063 | Focal brain injury | 8843471 |
| S063 | Focal brain injury | 8843548 |
| S063 | Focal brain injury | 8843562 |
| S064 | Epidural haemorrhage | 4320006 |
| S064 | Epidural haemorrhage | 4320008 |
| S064 | Epidural haemorrhage | 8520005 |
| S064 | Epidural haemorrhage | 8520016 |
| S064 | Epidural haemorrhage | 8843166 |
| S064 | Epidural haemorrhage | 8843176 |
| S064 | Epidural haemorrhage | 8843289 |
| S064 | Epidural haemorrhage | 8843290 |
| S064 | Epidural haemorrhage | 8843322 |
| S064 | Epidural haemorrhage | 8843330 |
| S064 | Epidural haemorrhage | 8843331 |
| S064 | Epidural haemorrhage | 8843420 |
| S064 | Epidural haemorrhage | 8843421 |
| S064 | Epidural haemorrhage | 8843544 |
| S064 | Epidural haemorrhage | 8843551 |
| S064 | Epidural haemorrhage | 8843554 |
| S064 | Epidural haemorrhage | 8843576 |
| S065 | Traumatic subdural haemorrhage | 4321013 |
| S065 | Traumatic subdural haemorrhage | 8520013 |
| S065 | Traumatic subdural haemorrhage | 8520015 |
| S065 | Traumatic subdural haemorrhage | 8843165 |
| S065 | Traumatic subdural haemorrhage | 8843175 |
| S065 | Traumatic subdural haemorrhage | 8843543 |
| S065 | Traumatic subdural haemorrhage | 8843553 |
| S065 | Traumatic subdural haemorrhage | 8843597 |
| S068 | Other intracranial injuries | 4321014 |
| S100 | Contusion of throat | 8830669 |
| S100 | Contusion of throat | 8830670 |
| S100 | Contusion of throat | 8832153 |
| S100 | Contusion of throat | 8833632 |
| S100 | Contusion of throat | 8833633 |
| S241 | Other and unspecified injuries of thoracic spinal cord | 8847086 |
| S273 | Other injuries of lung | 8838830 |
| S273 | Other injuries of lung | 8843214 |
| S273 | Other injuries of lung | 8843486 |
| S273 | Other injuries of lung | 8843487 |
| S273 | Other injuries of lung | 8843594 |
| S278 | Injury of other specified intrathoracic organs | 5193003 |
| S278 | Injury of other specified intrathoracic organs | 8843188 |
| S278 | Injury of other specified intrathoracic organs | 8843364 |
| S278 | Injury of other specified intrathoracic organs | 8843566 |
| S279 | Injury of unspecified intrathoracic organ | 8832484 |
| S279 | Injury of unspecified intrathoracic organ | 8843295 |
| S279 | Injury of unspecified intrathoracic organ | 8843296 |
| S279 | Injury of unspecified intrathoracic organ | 8844860 |
| S300 | Contusion of lower back and pelvis | 8833842 |
| S300 | Contusion of lower back and pelvis | 8836098 |
| S300 | Contusion of lower back and pelvis | 8837726 |
| S300 | Contusion of lower back and pelvis | 8837983 |
| S300 | Contusion of lower back and pelvis | 8839461 |
| S300 | Contusion of lower back and pelvis | 8840806 |
| S300 | Contusion of lower back and pelvis | 8840826 |
| S300 | Contusion of lower back and pelvis | 8840830 |
| S300 | Contusion of lower back and pelvis | 8840833 |
| S300 | Contusion of lower back and pelvis | 8840839 |
| S300 | Contusion of lower back and pelvis | 8840840 |
| S301 | Contusion of abdominal wall | 8836776 |
| S301 | Contusion of abdominal wall | 8836787 |
| S301 | Contusion of abdominal wall | 8839750 |
| S301 | Contusion of abdominal wall | 8839752 |
| S302 | Contusion of external genital organs | 8830625 |
| S302 | Contusion of external genital organs | 8830645 |
| S302 | Contusion of external genital organs | 8830687 |
| S302 | Contusion of external genital organs | 8830688 |
| S302 | Contusion of external genital organs | 8830732 |
| S302 | Contusion of external genital organs | 8830824 |
| S302 | Contusion of external genital organs | 8830825 |
| S302 | Contusion of external genital organs | 8835896 |
| S302 | Contusion of external genital organs | 8837502 |
| S302 | Contusion of external genital organs | 9224005 |
| S341 | Other injury of lumbar spinal cord | 8847142 |
| S361 | Injury of liver or gallbladder | 8638016 |
| S361 | Injury of liver or gallbladder | 8638017 |
| S361 | Injury of liver or gallbladder | 8843142 |
| S361 | Injury of liver or gallbladder | 8843143 |
| S361 | Injury of liver or gallbladder | 8843232 |
| S361 | Injury of liver or gallbladder | 8843234 |
| S361 | Injury of liver or gallbladder | 8843519 |
| S361 | Injury of liver or gallbladder | 8843520 |
| S369 | Injury of unspecified intra-abdominal organ | 8834673 |
| S369 | Injury of unspecified intra-abdominal organ | 8843351 |
| S369 | Injury of unspecified intra-abdominal organ | 8843560 |
| S390 | Injury of muscle and tendon of abdomen, lower back and pelvis | 8839786 |
| S390 | Injury of muscle and tendon of abdomen, lower back and pelvis | 8846320 |
| S400 | Contusion of shoulder and upper arm | 8831334 |
| S400 | Contusion of shoulder and upper arm | 8831348 |
| S400 | Contusion of shoulder and upper arm | 8833234 |
| S400 | Contusion of shoulder and upper arm | 8833238 |
| S400 | Contusion of shoulder and upper arm | 8833245 |
| S400 | Contusion of shoulder and upper arm | 8835556 |
| S400 | Contusion of shoulder and upper arm | 8845332 |
| S500 | Contusion of elbow | 8839271 |
| S500 | Contusion of elbow | 8839272 |
| S500 | Contusion of elbow | 8839281 |
| S501 | Contusion of other and unspecified parts of forearm | 8836599 |
| S501 | Contusion of other and unspecified parts of forearm | 8845361 |
| S700 | Contusion of hip | 8833738 |
| S701 | Contusion of thigh | 8837322 |
| S701 | Contusion of thigh | 8837334 |
| S701 | Contusion of thigh | 8837353 |
| S761 | Injury of quadriceps muscle and tendon | 8837357 |
| S762 | Injury of adductor muscle and tendon of thigh | 8837352 |
| S771 | Crushing injury of thigh | 8837282 |
| S800 | Contusion of knee | 8834429 |
| S800 | Contusion of knee | 8834431 |
| S800 | Contusion of knee | 8834444 |
| S800 | Contusion of knee | 8834446 |
| S800 | Contusion of knee | 8839234 |
| S800 | Contusion of knee | 9241017 |
| S801 | Contusion of other and unspecified parts of lower leg | 8831298 |
| S801 | Contusion of other and unspecified parts of lower leg | 8845229 |
| S898 | Other specified injuries of lower leg | 8846255 |
| S898 | Other specified injuries of lower leg | 8846330 |
| T001 | Superficial injuries involving thorax with abdomen, lower back and pelvis | 8832569 |
| T001 | Superficial injuries involving thorax with abdomen, lower back and pelvis | 8832656 |
| T009 | Multiple superficial injuries, unspecified | 8834306 |
| T009 | Multiple superficial injuries, unspecified | 8836511 |
| T009 | Multiple superficial injuries, unspecified | 8837018 |
| T009 | Multiple superficial injuries, unspecified | 8837028 |
| T009 | Multiple superficial injuries, unspecified | 8837065 |
| T090 | Superficial injury of trunk, level unspecified | 8838808 |
| T090 | Superficial injury of trunk, level unspecified | 8838883 |
| T090 | Superficial injury of trunk, level unspecified | 8845469 |
| T140 | Superficial injury of unspecified body region | 8831598 |
| T140 | Superficial injury of unspecified body region | 8837443 |
| T140 | Superficial injury of unspecified body region | 8837446 |
| T140 | Superficial injury of unspecified body region | 9249002 |
| T140 | Superficial injury of unspecified body region | 9249011 |
| T140 | Superficial injury of unspecified body region | 9249013 |
| T140 | Superficial injury of unspecified body region | 9249014 |
| T140 | Superficial injury of unspecified body region | 9249027 |
| T146 | Injury of muscles and tendons of unspecified body region | 9249009 |
| T810 | Haemorrhage and haematoma complicating a procedure, not elsewhere classified | 5258013 |
| T810 | Haemorrhage and haematoma complicating a procedure, not elsewhere classified | 8833916 |
| T810 | Haemorrhage and haematoma complicating a procedure, not elsewhere classified | 8837515 |
| T810 | Haemorrhage and haematoma complicating a procedure, not elsewhere classified | 8842779 |
| T810 | Haemorrhage and haematoma complicating a procedure, not elsewhere classified | 8844476 |
| T810 | Haemorrhage and haematoma complicating a procedure, not elsewhere classified | 8846238 |
| T810 | Haemorrhage and haematoma complicating a procedure, not elsewhere classified | 9983022 |
| T811 | Shock during or resulting from a procedure, not elsewhere classified | 8844477 |
| T811 | Shock during or resulting from a procedure, not elsewhere classified | 9980002 |
| T905 | Sequelae of intracranial injury | 9070010 |
