# Supplementary material for: Assessment of bleeding in patients with disseminated intravascular coagulation after receiving surgery and recombinant human soluble thrombomodulin: A cohort study using a database
Source: PLoS One. 2018 Oct 8;13(10):e0205146. doi: 10.1371/journal.pone.0205146 (PMC6175500; doi:10.1371/journal.pone.0205146)
Supplement: S2 Table — (DOCX) [file pone.0205146.s006.docx]

**S2 Table. Usage ratio of the most frequently used drugs in the non-rTM treatment group**

| Other treatments | Commercial and generic names of the most frequently used drugs | Usage ratio (%) |
| --- | --- | --- |
| Antithrombin III | Neuart Intravenous 500 U | 11.4 |
| Heparin | Heparin sodium 5,000 U/5 mL for injection MOCHIDA | 34.8 |
| Platelet concentrate | Irradiated platelet concentrate, Leukocytes Reduced, NISSEKI (Ir-PC-LR) | 22.8 |
| Fresh frozen plasma | Fresh frozen plasma, Leukocytes reduced, NISSEKI 120 (FFP-LR120) | 22.4 |
| Serine protease inhibitors | REMINARON | 16.4 |
| Xa-factor | ORGARAN Intravenous 1250 U | 3.4 |
| Fibrinogen concentrate | Fibrinogen HT Intravenous 1g “JB (Japan Blood Products Organization)” | 0.5 |
