# Supplementary material for: Assessment of bleeding in patients with disseminated intravascular coagulation after receiving surgery and recombinant human soluble thrombomodulin: A cohort study using a database
Source: PLoS One. 2018 Oct 8;13(10):e0205146. doi: 10.1371/journal.pone.0205146 (PMC6175500; doi:10.1371/journal.pone.0205146)
Supplement: S3 Table — aIncludes diseases that seem to be severe (e.g., liver cirrhosis), but as defined in the previous study (Quan et al. Med Care. 2005;43:1130–9.) bWhen the test was performed a few times on the same day, we adopted the average. cWhen the test was performed a few times on the same day, we adopted the minimum (sensitivity analysis). dWhen the test was performed a few times on the same day, we adopted the maximum (sensitivity analysis). rTM, recombinant thrombomodulin; DIC, disseminated intravascular coagulation; FDP, fibrinogen/fibrin degradation products. (DOCX) [file pone.0205146.s007.docx]

**S3 Table.** **Demographic and clinical baseline characteristics of patients who underwent hepatic, biliary, or pancreatic surgery**

| **Item** | **Classification** |  | **Before matching** | | | **After matching** | | |
| --- | --- | --- | --- | --- | --- | --- | --- | --- |
|  |  |  | **rTM group** | **Non-rTM group** | **Standardized difference %** | **rTM group** | **Non-rTM group** | **Standardized difference %** |
|  |  |  | **N = 672** | **N = 1464** |  | **N = 568** | **N = 568** |  |
| Sex, n (%) | Male |  | 418 (62.2) | 895 (61.1) | 2.2 | 353 (62.1) | 336 (59.2) | 6.1 |
|  | Female |  | 254 (37.8) | 569 (38.9) | - | 215 (37.9) | 232 (40.8) | - |
| Age, years | N |  | 672 | 1464 | - | 568 | 568 | - |
|  | Median (Minimum/ maximum) |  | 78.0  (33/102) | 75.0  (18/100) | - | 78.0  (33/99) | 78.0  (23/100) | - |
| Concomitant drugs, n (%) | Catecholamine | No | 350 (52.1) | 779 (53.2) | 2.3 | 313 (55.1) | 325 (57.2) | 4.3 |
|  |  | Yes | 322 (47.9) | 685 (46.8) | - | 255 (44.9) | 243 (42.8) | - |
|  | Antibiotics | No | 3 (0.4) | 19 (1.3) | 9.2 | 2 (0.4) | 2 (0.4) | 0.0 |
|  |  | Yes | 669 (99.6) | 1445 (98.7) | - | 566 (99.6) | 566 (99.6) | - |
|  | Antifungals | No | 663 (98.7) | 1460 (99.7) | 11.9 | 563 (99.1) | 564 (99.3) | 2.0 |
|  |  | Yes | 9 (1.3) | 4 (0.3) | - | 5 (0.9) | 4 (0.7) | - |
|  | Other drugs for DIC treatment | No | 193 (28.7) | 0 (0.0) | 89.8 | 173 (30.5) | 0 (0.0) | 93.6 |
|  |  | Yes | 479 (71.3) | 1464 (100.0) | - | 395 (69.5) | 568 (100.0) | - |
|  | Steroids | No | 571 (85.0) | 1121 (76.6) | 21.4 | 484 (85.2) | 499 (87.9) | 7.7 |
|  |  | Yes | 101 (15.0) | 343 (23.4) | - | 84 (14.8) | 69 (12.1) | - |
|  | Neutrophil elastase inhibitors (Sivelestat) | No | 638 (94.9) | 1424 (97.3) | 12.0 | 546 (96.1) | 548 (96.5) | 1.9 |
|  |  | Yes | 34 (5.1) | 40 (2.7) | - | 22 (3.9) | 20 (3.5) | - |
|  | Immunoglobulins | No | 531 (79.0) | 1339 (91.5) | 35.6 | 469 (82.6) | 470 (82.7) | 0.5 |
|  |  | Yes | 141 (21.0) | 125 (8.5) | - | 99 (17.4) | 98 (17.3) | - |
| Complications, n (%) | Myocardial infarction | No | 664 (98.8) | 1442 (98.5) | 2.7 | 561 (98.8) | 561 (98.8) | 0.0 |
|  |  | Yes | 8 (1.2) | 22 (1.5) | - | 7 (1.2) | 7 (1.2) | - |
|  | Congestive heart failure | No | 617 (91.8) | 1356 (92.6) | 3.0 | 521 (91.7) | 519 (91.4) | 1.3 |
|  |  | Yes | 55 (8.2) | 108 (7.4) | - | 47 (8.3) | 49 (8.6) | - |
|  | Peripheral vascular disease | No | 664 (98.8) | 1444 (98.6) | 1.6 | 560 (98.6) | 560 (98.6) | 0.0 |
|  |  | Yes | 8 (1.2) | 20 (1.4) | - | 8 (1.4) | 8 (1.4) | - |
|  | Cerebral vascular disease | No | 630 (93.8) | 1385 (94.6) | 3.6 | 534 (94.0) | 534 (94.0) | 0.0 |
|  |  | Yes | 42 (6.3) | 79 (5.4) | - | 34 (6.0) | 34 (6.0) | - |
|  | Dementia | No | 645 (96.0) | 1434 (98.0) | 11.5 | 543 (95.6) | 543 (95.6) | 0.0 |
|  |  | Yes | 27 (4.0) | 30 (2.0) | - | 25 (4.4) | 25 (4.4) | - |
|  | Chronic lung disease | No | 653 (97.2) | 1419 (96.9) | 1.5 | 552 (97.2) | 548 (96.5) | 4.0 |
|  |  | Yes | 19 (2.8) | 45 (3.1) | - | 16 (2.8) | 20 (3.5) | - |
|  | Collagen disease | No | 669 (99.6) | 1450 (99.0) | 6.1 | 565 (99.5) | 564 (99.3) | 2.2 |
|  |  | Yes | 3 (0.4) | 14 (1.0) | - | 3 (0.5) | 4 (0.7) | - |
|  | Peptic ulcer | No | 614 (91.4) | 1303 (89.0) | 8.0 | 516 (90.8) | 509 (89.6) | 4.2 |
|  |  | Yes | 58 (8.6) | 161 (11.0) | - | 52 (9.2) | 59 (10.4) | - |
|  | Mild liver disease^a^ | No | 626 (93.2) | 1226 (83.7) | 29.8 | 526 (92.6) | 541 (95.2) | 11.1 |
|  |  | Yes | 46 (6.8) | 238 (16.3) | - | 42 (7.4) | 27 (4.8) | - |
|  | Diabetes | No | 579 (86.2) | 1175 (80.3) | 15.8 | 485 (85.4) | 495 (87.1) | 5.1 |
|  |  | Yes | 93 (13.8) | 289 (19.7) | - | 83 (14.6) | 73 (12.9) | - |
|  | Hemiplegia | No | 670 (99.7) | 1463 (99.9) | 5.4 | 566 (99.6) | 567 (99.8) | 3.4 |
|  |  | Yes | 2 (0.3) | 1 (0.1) | - | 2 (0.4) | 1 (0.2) | - |
|  | Renal dysfunction | No | 635 (94.5) | 1423 (97.2) | 13.6 | 542 (95.4) | 547 (96.3) | 4.4 |
|  |  | Yes | 37 (5.5) | 41 (2.8) | - | 26 (4.6) | 21 (3.7) | - |
|  | Diabetes mellitus with chronic complications | No | 645 (96.0) | 1432 (97.8) | 10.6 | 549 (96.7) | 544 (95.8) | 4.6 |
|  |  | Yes | 27 (4.0) | 32 (2.2) | - | 19 (3.3) | 24 (4.2) | - |
|  | Solid cancer, leukemia, lymphoma | No | 459 (68.3) | 823 (56.2) | 25.1 | 379 (66.7) | 395 (69.5) | 6.0 |
|  |  | Yes | 213 (31.7) | 641 (43.8) | - | 189 (33.3) | 173 (30.5) | - |
|  | Moderate to high liver dysfunction | No | 661 (98.4) | 1412 (96.4) | 12.1 | 558 (98.2) | 564 (99.3) | 9.6 |
|  |  | Yes | 11 (1.6) | 52 (3.6) | - | 10 (1.8) | 4 (0.7) | - |
|  | Metastatic solid tumors | No | 621 (92.4) | 1337 (91.3) | 4.0 | 523 (92.1) | 524 (92.3) | 0.7 |
|  |  | Yes | 51 (7.6) | 127 (8.7) | - | 45 (7.9) | 44 (7.7) | - |
|  | AIDS・HIV | No | 671 (99.9) | 1464 (100.0) | 5.5 | 568 (100.0) | 568 (100.0) | - |
|  |  | Yes | 1 (0.1) | 0 (0.0) | - | 0 (0.0) | 0 (0.0) | - |
| Treatments, n (%) | Ventilators | No | 592 (88.1) | 1405 (96.0) | 29.4 | 519 (91.4) | 528 (93.0) | 5.9 |
|  |  | Yes | 80 (11.9) | 59 (4.0) | - | 49 (8.6) | 40 (7.0) | - |
|  | Dialysis | No | 607 (90.3) | 1437 (98.2) | 34.1 | 541 (95.2) | 544 (95.8) | 2.6 |
|  |  | Yes | 65 (9.7) | 27 (1.8) | - | 27 (4.8) | 24 (4.2) | - |
|  | Extracorporeal membrane oxygenation | No | 672 (100.0) | 1464 (100.0) | - | 568 (100.0) | 568 (100.0) | - |
|  |  | Yes | 0 (0.0) | 0 (0.0) | - | 0 (0.0) | 0 (0.0) | - |
|  | Intra-aortic balloon pumping | No | 672 (100.0) | 1464 (100.0) | - | 568 (100.0) | 568 (100.0) | - |
|  |  | Yes | 0 (0.0) | 0 (0.0) | - | 0 (0.0) | 0 (0.0) | - |
|  | Central venous catheterization | No | 669 (99.6) | 1450 (99.0) | 6.1 | 565 (99.5) | 565 (99.5) | 0.0 |
|  |  | Yes | 3 (0.4) | 14 (1.0) | - | 3 (0.5) | 3 (0.5) | - |
|  | Pleural effusion | No | 455 (67.7) | 1334 (91.1) | 60.5 | 437 (76.9) | 455 (80.1) | 7.7 |
|  |  | Yes | 217 (32.3) | 130 (8.9) | - | 131 (23.1) | 113 (19.9) | - |
|  | Blood purification therapy | No | 607 (90.3) | 1437 (98.2) | 34.1 | 541 (95.2) | 544 (95.8) | 2.6 |
|  |  | Yes | 65 (9.7) | 27 (1.8) | - | 27 (4.8) | 24 (4.2) | - |
|  | Coagulation blood test | No | 33 (4.9) | 298 (20.4) | 47.8 | 33 (5.8) | 24 (4.2) | 7.3 |
|  |  | Yes | 639 (95.1) | 1166 (79.6) | - | 535 (94.2) | 544 (95.8) | - |
| Transfusion | Red blood cell transfusion | No | 587 (87.4) | 1180 (80.6) | 18.5 | 501 (88.2) | 505 (88.9) | 2.2 |
|  |  | Yes | 85 (12.6) | 284 (19.4) | - | 67 (11.8) | 63 (11.1) | - |
|  | Whole blood transfusion | No | 672 (100.0) | 1464 (100.0) | - | 568 (100.0) | 568 (100.0) | - |
|  |  | Yes | 0 (0.0) | 0 (0.0) | - | 0 (0.0) | 0 (0.0) | - |
| Number of beds in medical facilities, n (%) | <200 beds |  | 16 (2.4) | 51 (3.5) | 6.5 | 14 (2.5) | 21 (3.7) | 7.1 |
|  | ≥200, <500 |  | 378 (56.3) | 716 (48.9) | 14.7 | 315 (55.5) | 312 (54.9) | 1.1 |
|  | ≥500 |  | 278 (41.4) | 697 (47.6) | 12.6 | 239 (42.1) | 235 (41.4) | 1.4 |
| Platelets  (average) | N |  | 43 | 64 | - | 35 | 32 | - |
|  | Median |  | 7.40 | 14.90 | - | 7.40 | 12.70 | - |
| Creatinine  (average^b^) | N |  | 43 | 64 | - | 35 | 32 | - |
|  | Median |  | 1.320 | 0.820 | - | 1.070 | 0.875 | - |
| Total bilirubin  (average^b^) | N |  | 38 | 64 | - | 30 | 32 | - |
|  | Median |  | 1.800 | 1.635 | - | 1.800 | 2.165 | - |
| Direct bilirubin  (average^b^) | N |  | 27 | 55 | - | 21 | 27 | - |
|  | Median |  | 0.900 | 0.760 | - | 1.000 | 1.750 | - |
| FDP/D-dimer  (average) | N |  | 8 | 14 | - | 5 | 10 | - |
|  | Median |  | 8.800 | 9.450 | - | 9.100 | 10.150 | - |
| Platelets  (minimum^c^) | N |  | 43 | 64 | - | 35 | 32 | - |
|  | Median |  | 7.40 | 14.90 | - | 7.40 | 12.70 | - |
| Creatinine  (minimum^c^) | N |  | 43 | 64 | - | 35 | 32 | - |
|  | Median |  | 1.290 | 0.820 | - | 1.070 | 0.875 | - |
| Total bilirubin  (minimum^c^) | N |  | 38 | 64 | - | 30 | 32 | - |
|  | Median |  | 1.800 | 1.635 | - | 1.800 | 2.165 | - |
| Direct bilirubin  (minimum^c^) | n |  | 27 | 55 | - | 21 | 27 | - |
|  | Median |  | 0.900 | 0.760 | - | 1.000 | 1.750 | - |
| FDP/D-dimer  (minimum^c^) | N |  | 8 | 14 | - | 5 | 10 | - |
|  | Median |  | 8.800 | 9.450 | - | 9.100 | 10.150 | - |
| Platelets  (maximum^d^) | N |  | 43 | 64 | - | 35 | 32 | - |
|  | Median |  | 8.20 | 14.90 | - | 7.40 | 12.70 | - |
| Creatinine  (maximum^d^) | N |  | 43 | 64 | - | 35 | 32 | - |
|  | Median |  | 1.400 | 0.820 | - | 1.080 | 0.875 | - |
| Total bilirubin  (maximum^d^) | N |  | 38 | 64 | - | 30 | 32 | - |
|  | Median |  | 1.800 | 1.635 | - | 1.800 | 2.165 | - |
| Direct bilirubin  (maximum^d^) | N |  | 27 | 55 | - | 21 | 27 | - |
|  | Median |  | 0.900 | 0.760 | - | 1.000 | 1.750 | - |
| FDP/D-dimer  (maximum^d^) | N |  | 8 | 14 | - | 5 | 10 | - |
|  | Median |  | 8.800 | 9.450 | - | 9.100 | 10.150 | - |

^a^Includes diseases that seem to be severe (e.g., liver cirrhosis), but as defined in the previous study (Quan H, Med Care, 2005, 43, 1130)

^b^When there were repeated test results, we adopted the average.

^c^When there were repeated test results, we adopted the minimum.

^d^When there were repeated test results, we adopted the maximum.

rTM, recombinant thrombomodulin; DIC, disseminated intravascular coagulation; FDP, fibrinogen/fibrin degradation products
