# Supplementary material for: Assessment of bleeding in patients with disseminated intravascular coagulation after receiving surgery and recombinant human soluble thrombomodulin: A cohort study using a database
Source: PLoS One. 2018 Oct 8;13(10):e0205146. doi: 10.1371/journal.pone.0205146 (PMC6175500; doi:10.1371/journal.pone.0205146)
Supplement: S4 Table — aIncludes diseases that seem to be severe (e.g., liver cirrhosis), but as defined in the previous study (Quan et al. Med Care. 2005;43:1130–9.) bWhen the test was performed a few times on the same day, we adopted the average. cWhen the test was performed a few times on the same day, we adopted the minimum (sensitivity analysis). dWhen the test was performed a few times on the same day, we adopted the maximum (sensitivity analysis). rTM, recombinant thrombomodulin; DIC, disseminated intravascular coagulation; FDP, fibrinogen/fibrin degradation products. (DOCX) [file pone.0205146.s008.docx]

**S4 Table.** **Demographic and clinical baseline characteristics of patients who underwent other gastrointestinal surgery**

| **Item** | **Classification** |  | **Before matching** | | | **After matching** | | |
| --- | --- | --- | --- | --- | --- | --- | --- | --- |
|  |  |  | **rTM group** | **non-rTM group** | **Standardized difference %** | **rTM group** | **non-rTM group** | **Standardized difference %** |
|  |  |  | **N = 956** | **N = 1278** |  | **N = 614** | **N = 614** |  |
| Sex, n (%) | Male |  | 539 (56.4) | 780 (61.0) | 9.5 | 361 (58.8) | 345 (56.2) | 5.3 |
|  | Female |  | 417 (43.6) | 498 (39.0) | - | 253 (41.2) | 269 (43.8) | - |
| Age, years | n |  | 956 | 1278 | - | 614 | 614 | - |
|  | Median (Minimum/ maximum) |  | 77.0 (21/100) | 74.5  (23/103) | - | 77.0  (21/99) | 77.0 (23/103) | - |
| Concomitant drugs, n (%) | Catecholamine | No | 205 (21.4) | 437 (34.2) | 28.7 | 167 (27.2) | 167 (27.2) | 0.0 |
|  |  | Yes | 751 (78.6) | 841 (65.8) | - | 447 (72.8) | 447 (72.8) | - |
|  | Antibiotics | No | 1 (0.1) | 36 (2.8) | 22.8 | 1 (0.2) | 0 (0.0) | 5.7 |
|  |  | Yes | 955 (99.9) | 1242 (97.2) | - | 613 (99.8) | 614 (100.0) | - |
|  | Antifungals | No | 905 (94.7) | 1252 (98.0) | 17.6 | 593 (96.6) | 591 (96.3) | 1.8 |
|  |  | Yes | 51 (5.3) | 26 (2.0) | - | 21 (3.4) | 23 (3.7) | - |
|  | Other drugs for DIC treatment | No | 100 (10.5) | 0 (0.0) | 48.3 | 90 (14.7) | 0 (0.0) | 58.6 |
|  |  | Yes | 856 (89.5) | 1278 (100.0) | - | 524 (85.3) | 614 (100.0) | - |
|  | Steroids | No | 716 (74.9) | 1021 (79.9) | 12.0 | 479 (78.0) | 488 (79.5) | 3.6 |
|  |  | Yes | 240 (25.1) | 257 (20.1) | - | 135 (22.0) | 126 (20.5) | - |
|  | Neutrophil elastase inhibitors (Sivelestat) | No | 677 (70.8) | 1045 (81.8) | 26.0 | 470 (76.5) | 464 (75.6) | 2.3 |
|  |  | Yes | 279 (29.2) | 233 (18.2) | - | 144 (23.5) | 150 (24.4) | - |
|  | Immunoglobulins | No | 485 (50.7) | 963 (75.4) | 52.7 | 367 (59.8) | 369 (60.1) | 0.7 |
|  |  | Yes | 471 (49.3) | 315 (24.6) | - | 247 (40.2) | 245 (39.9) | - |
| Complications, n (%) | Myocardial infarction | No | 945 (98.8) | 1261 (98.7) | 1.6 | 608 (99.0) | 607 (98.9) | 1.6 |
|  |  | Yes | 11 (1.2) | 17 (1.3) | - | 6 (1.0) | 7 (1.1) | - |
|  | Congestive heart failure | No | 875 (91.5) | 1151 (90.1) | 5.1 | 568 (92.5) | 558 (90.9) | 5.9 |
|  |  | Yes | 81 (8.5) | 127 (9.9) | - | 46 (7.5) | 56 (9.1) | - |
|  | Peripheral vascular disease | No | 928 (97.1) | 1241 (97.1) | 0.2 | 594 (96.7) | 595 (96.9) | 0.9 |
|  |  | Yes | 28 (2.9) | 37 (2.9) | - | 20 (3.3) | 19 (3.1) | - |
|  | Cerebral vascular disease | No | 914 (95.6) | 1209 (94.6) | 4.7 | 585 (95.3) | 586 (95.4) | 0.8 |
|  |  | Yes | 42 (4.4) | 69 (5.4) | - | 29 (4.7) | 28 (4.6) | - |
|  | Dementia | No | 938 (98.1) | 1261 (98.7) | 4.4 | 605 (98.5) | 604 (98.4) | 1.3 |
|  |  | Yes | 18 (1.9) | 17 (1.3) | - | 9 (1.5) | 10 (1.6) | - |
|  | Chronic lung disease | No | 928 (97.1) | 1210 (94.7) | 12.0 | 591 (96.3) | 584 (95.1) | 5.6 |
|  |  | Yes | 28 (2.9) | 68 (5.3) | - | 23 (3.7) | 30 (4.9) | - |
|  | Collagen disease | No | 948 (99.2) | 1265 (99.0) | 1.9 | 610 (99.3) | 608 (99.0) | 3.6 |
|  |  | Yes | 8 (0.8) | 13 (1.0) | - | 4 (0.7) | 6 (1.0) | - |
|  | Peptic ulcer | No | 853 (89.2) | 1119 (87.6) | 5.2 | 546 (88.9) | 541 (88.1) | 2.6 |
|  |  | Yes | 103 (10.8) | 159 (12.4) | - | 68 (11.1) | 73 (11.9) | - |
|  | Mild liver disease^a^ | No | 919 (96.1) | 1159 (90.7) | 22.1 | 587 (95.6) | 592 (96.4) | 4.2 |
|  |  | Yes | 37 (3.9) | 119 (9.3) | - | 27 (4.4) | 22 (3.6) | - |
|  | Diabetes | No | 861 (90.1) | 1140 (89.2) | 2.8 | 546 (88.9) | 534 (87.0) | 6.0 |
|  |  | Yes | 95 (9.9) | 138 (10.8) | - | 68 (11.1) | 80 (13.0) | - |
|  | Hemiplegia | No | 954 (99.8) | 1276 (99.8) | 1.2 | 614 (100.0) | 614 (100.0) | - |
|  |  | Yes | 2 (0.2) | 2 (0.2) | - | 0 (0.0) | 0 (0.0) | - |
|  | Renal dysfunction | No | 883 (92.4) | 1211 (94.8) | 9.8 | 575 (93.6) | 574 (93.5) | 0.7 |
|  |  | Yes | 73 (7.6) | 67 (5.2) | - | 39 (6.4) | 40 (6.5) | - |
|  | Diabetes mellitus with chronic complications | No | 944 (98.7) | 1248 (97.7) | 8.2 | 603 (98.2) | 603 (98.2) | 0.0 |
|  |  | Yes | 12 (1.3) | 30 (2.3) | - | 11 (1.8) | 11 (1.8) | - |
|  | Solid cancer, leukemia, lymphoma | No | 891 (93.2) | 1133 (88.7) | 15.9 | 566 (92.2) | 564 (91.9) | 1.2 |
|  |  | Yes | 65 (6.8) | 145 (11.3) | - | 48 (7.8) | 50 (8.1) | - |
|  | Moderate to high liver dysfunction | No | 943 (98.6) | 1224 (95.8) | 17.5 | 604 (98.4) | 606 (98.7) | 2.7 |
|  |  | Yes | 13 (1.4) | 54 (4.2) | - | 10 (1.6) | 8 (1.3) | - |
|  | Metastatic solid tumors | No | 889 (93.0) | 1136 (88.9) | 14.3 | 567 (92.3) | 566 (92.2) | 0.6 |
|  |  | Yes | 67 (7.0) | 142 (11.1) | - | 47 (7.7) | 48 (7.8) | - |
|  | AIDS・HIV | No | 955 (99.9) | 1278 (100.0) | 4.6 | 614 (100.0) | 614 (100.0) | - |
|  |  | Yes | 1 (0.1) | 0 (0.0) | - | 0 (0.0) | 0 (0.0) | - |
| Treatments, n (%) | Ventilators | No | 495 (51.8) | 1056 (82.6) | 69.6 | 432 (70.4) | 440 (71.7) | 2.9 |
|  |  | Yes | 461 (48.2) | 222 (17.4) | - | 182 (29.6) | 174 (28.3) | - |
|  | Dialysis | No | 714 (74.7) | 1170 (91.5) | 46.2 | 522 (85.0) | 522 (85.0) | 0.0 |
|  |  | Yes | 242 (25.3) | 108 (8.5) | - | 92 (15.0) | 92 (15.0) | - |
|  | Extracorporeal membrane oxygenation | No | 956 (100.0) | 1278 (100.0) | - | 614 (100.0) | 614 (100.0) | - |
|  |  | Yes | 0 (0.0) | 0 (0.0) | - | 0 (0.0) | 0 (0.0) | - |
|  | Intra-aortic Balloon Pumping | No | 956 (100.0) | 1278 (100.0) | - | 614 (100.0) | 614 (100.0) | - |
|  |  | Yes | 0 (0.0) | 0 (0.0) | - | 0 (0.0) | 0 (0.0) | - |
|  | Central venous catheterization | No | 948 (99.2) | 1263 (98.8) | 3.4 | 608 (99.0) | 609 (99.2) | 1.7 |
|  |  | Yes | 8 (0.8) | 15 (1.2) | - | 6 (1.0) | 5 (0.8) | - |
|  | Pleural effusion | No | 403 (42.2) | 1009 (79.0) | 81.3 | 370 (60.3) | 377 (61.4) | 2.3 |
|  |  | Yes | 553 (57.8) | 269 (21.0) | - | 244 (39.7) | 237 (38.6) | - |
|  | Blood purification therapy | No | 714 (74.7) | 1170 (91.5) | 46.2 | 522 (85.0) | 522 (85.0) | 0.0 |
|  |  | Yes | 242 (25.3) | 108 (8.5) | - | 92 (15.0) | 92 (15.0) | - |
|  | Coagulation blood test | No | 19 (2.0) | 143 (11.2) | 37.7 | 19 (3.1) | 19 (3.1) | 0.0 |
|  |  | Yes | 937 (98.0) | 1135 (88.8) | - | 595 (96.9) | 595 (96.9) | - |
| Transfusion, n (%) | Red blood cell transfusion | No | 508 (53.1) | 707 (55.3) | 4.4 | 344 (56.0) | 356 (58.0) | 3.9 |
|  |  | Yes | 448 (46.9) | 571 (44.7) | - | 270 (44.0) | 258 (42.0) | - |
|  | Whole blood transfusion | No | 956 (100.0) | 1278 (100.0) | - | 614 (100.0) | 614 (100.0) | - |
|  |  | Yes | 0 (0.0) | 0 (0.0) | - | 0 (0.0) | 0 (0.0) | - |
| Number of beds in medical facilities, n (%) | <200 beds |  | 20 (2.1) | 61 (4.8) | 14.8 | 13 (2.1) | 35 (5.7) | 18.6 |
|  | ≥200, <500 |  | 592 (61.9) | 751 (58.8) | 6.5 | 399 (65.0) | 368 (59.9) | 10.4 |
|  | ≥500 |  | 344 (36.0) | 466 (36.5) | 1.0 | 202 (32.9) | 211 (34.4) | 3.1 |
| Platelets  (average^b^) | n |  | 77 | 43 | - | 51 | 20 | - |
|  | Median |  | 15.40 | 18.50 | - | 15.30 | 20.38 | - |
| Creatinine  (average) | n |  | 77 | 43 | - | 51 | 20 | - |
|  | Median |  | 0.990 | 0.890 | - | 0.950 | 0.950 | - |
| Total bilirubin  (average^b^) | n |  | 73 | 42 | - | 47 | 20 | - |
|  | Median |  | 0.700 | 0.700 | - | 0.750 | 0.680 | - |
| Direct bilirubin  (average^b^) | n |  | 37 | 21 | - | 25 | 7 | - |
|  | Median |  | 0.220 | 0.100 | - | 0.220 | 0.300 | - |
| FDP/D-dimer  (average^b^) | n |  | 29 | 10 | - | 16 | 4 | - |
|  | Median |  | 6.200 | 4.050 | - | 8.400 | 4.050 | - |
| Platelets  (minimum^c^) | n |  | 77 | 43 | - | 51 | 20 | - |
|  | Median |  | 14.20 | 16.10 | - | 14.90 | 19.90 | - |
| Creatinine  (minimum^c^) | n |  | 77 | 43 | - | 51 | 20 | - |
|  | Median |  | 0.950 | 0.890 | - | 0.890 | 0.935 | - |
| Total bilirubin  (minimum^c^) | n |  | 73 | 42 | - | 47 | 20 | - |
|  | Median |  | 0.670 | 0.700 | - | 0.750 | 0.680 | - |
| Direct bilirubin  (minimum^c^) | n |  | 37 | 21 | - | 25 | 7 | - |
|  | Median |  | 0.220 | 0.100 | - | 0.220 | 0.300 | - |
| FDP/D-dimer  (minimum^d^) | n |  | 29 | 10 | - | 16 | 4 | - |
|  | Median |  | 6.200 | 4.050 | - | 8.400 | 4.050 | - |
| Platelets  (maximum^d^) | n |  | 77 | 43 | - | 51 | 20 | - |
|  | Median |  | 16.30 | 19.30 | - | 16.30 | 23.90 | - |
| Creatinine  (maximum^d^) | n |  | 77 | 43 | - | 51 | 20 | - |
|  | Median |  | 0.990 | 0.900 | - | 0.950 | 0.970 | - |
| Total bilirubin  (maximum^d^) | n |  | 73 | 42 | - | 47 | 20 | - |
|  | Median |  | 0.730 | 0.700 | - | 0.750 | 0.750 | - |
| Direct bilirubin  (maximum^d^) | n |  | 37 | 21 | - | 25 | 7 | - |
|  | Median |  | 0.220 | 0.100 | - | 0.220 | 0.300 | - |
| FDP/D-dimer  (maximum^d^) | n |  | 29 | 10 | - | 16 | 4 | - |
|  | Median |  | 6.200 | 4.050 | - | 8.400 | 4.050 | - |

^a^Includes diseases that seem to be severe (e.g., liver cirrhosis), but as defined in the previous study (Quan H, Med Care, 2005, 43, 1130)

^b^When there were repeated test results, we adopted the average.

^c^When there were repeated test results, we adopted the minimum.

^d^When there were repeated test results, we adopted the maximum.

rTM, recombinant thrombomodulin; DIC, disseminated intravascular coagulation; FDP, fibrinogen/fibrin degradation products; SD, standard deviation
