# Supplementary material for: Assessment of bleeding in patients with disseminated intravascular coagulation after receiving surgery and recombinant human soluble thrombomodulin: A cohort study using a database
Source: PLoS One. 2018 Oct 8;13(10):e0205146. doi: 10.1371/journal.pone.0205146 (PMC6175500; doi:10.1371/journal.pone.0205146)
Supplement: S5 Table — aIncludes diseases that seem to be severe (e.g., liver cirrhosis), but as defined in the previous study (Quan et al. Med Care. 2005;43:1130–9.) bWhen the test was performed a few times on the same day, we adopted the average. cWhen the test was performed a few times on the same day, we adopted the minimum (sensitivity analysis).dWhen the test was performed a few times on the same day, we adopted the maximum (sensitivity analysis). rTM, recombinant thrombomodulin; DIC, disseminated intravascular coagulation; FDP, fibrinogen/fibrin degradation products. (DOCX) [file pone.0205146.s009.docx]

**S5 Table.** **Demographic and clinical baseline characteristics of patients who underwent cardiac/cardiovascular surgery**

| **Item** | **Classification** |  | **Before matching** | | | **After matching** | | |
| --- | --- | --- | --- | --- | --- | --- | --- | --- |
|  |  |  | **rTM group** | **non-rTM group** | **Standardized difference %** | **rTM group** | **non-rTM group** | **Standardized difference %** |
|  |  |  | **N = 523** | **N = 1391** |  | **N = 439** | **N = 439** |  |
| Sex, n (%) | Male |  | 295 (56.4) | 874 (62.8) | 13.1 | 255 (58.1) | 245 (55.8) | 4.6 |
|  | Female |  | 228 (43.6) | 517 (37.2) | - | 184 (41.9) | 194 (44.2) | - |
| Age, years | n |  | 523 | 1391 | - | 439 | 439 | - |
|  | Median  (Minimum/ maximum) |  | 73.0  (21/100) | 73.0 (18/100) | - | 73.0  (21/97) | 75.0 (18/100) | - |
| Concomitant drugs, n (%) | Catecholamine | No | 210 (40.2) | 539 (38.7) | 2.9 | 185 (42.1) | 192 (43.7) | 3.2 |
|  |  | Yes | 313 (59.8) | 852 (61.3) | - | 254 (57.9) | 247 (56.3) | - |
|  | Antibiotics | No | 49 (9.4) | 195 (14.0) | 14.5 | 49 (11.2) | 39 (8.9) | 7.6 |
|  |  | Yes | 474 (90.6) | 1196 (86.0) | - | 390 (88.8) | 400 (91.1) | - |
|  | Antifungals | No | 460 (88.0) | 1350 (97.1) | 35.1 | 402 (91.6) | 404 (92.0) | 1.7 |
|  |  | Yes | 63 (12.0) | 41 (2.9) | - | 37 (8.4) | 35 (8.0) | - |
|  | Other drugs for DIC treatment | No | 120 (22.9) | 0 (0.0) | 77.2 | 104 (23.7) | 0 (0.0) | 78.8 |
|  |  | Yes | 403 (77.1) | 1391 (100.0) | - | 335 (76.3) | 439 (100.0) | - |
|  | Steroids | No | 301 (57.6) | 955 (68.7) | 23.2 | 267 (60.8) | 283 (64.5) | 7.5 |
|  |  | Yes | 222 (42.4) | 436 (31.3) | - | 172 (39.2) | 156 (35.5) | - |
|  | Neutrophil elastase inhibitors (Sivelestat) | No | 438 (83.7) | 1247 (89.6) | 17.4 | 382 (87.0) | 382 (87.0) | 0.0 |
|  |  | Yes | 85 (16.3) | 144 (10.4) | - | 57 (13.0) | 57 (13.0) | - |
|  | Immunoglobulins | No | 428 (81.8) | 1282 (92.2) | 31.1 | 369 (84.1) | 372 (84.7) | 1.9 |
|  |  | Yes | 95 (18.2) | 109 (7.8) | - | 70 (15.9) | 67 (15.3) | - |
| Complications, n (%) | Myocardial infarction | No | 488 (93.3) | 1227 (88.2) | 17.7 | 405 (92.3) | 408 (92.9) | 2.6 |
|  |  | Yes | 35 (6.7) | 164 (11.8) | - | 34 (7.7) | 31 (7.1) | - |
|  | Congestive heart failure | No | 421 (80.5) | 952 (68.4) | 27.9 | 347 (79.0) | 352 (80.2) | 2.8 |
|  |  | Yes | 102 (19.5) | 439 (31.6) | - | 92 (21.0) | 87 (19.8) | - |
|  | Peripheral vascular disease | No | 490 (93.7) | 1116 (80.2) | 40.8 | 409 (93.2) | 412 (93.8) | 2.8 |
|  |  | Yes | 33 (6.3) | 275 (19.8) | - | 30 (6.8) | 27 (6.2) | - |
|  | Cerebral vascular disease | No | 483 (92.4) | 1243 (89.4) | 10.4 | 403 (91.8) | 398 (90.7) | 4.0 |
|  |  | Yes | 40 (7.6) | 148 (10.6) | - | 36 (8.2) | 41 (9.3) | - |
|  | Dementia | No | 515 (98.5) | 1373 (98.7) | 2.0 | 432 (98.4) | 429 (97.7) | 5.0 |
|  |  | Yes | 8 (1.5) | 18 (1.3) | - | 7 (1.6) | 10 (2.3) | - |
|  | Chronic lung disease | No | 499 (95.4) | 1318 (94.8) | 3.0 | 418 (95.2) | 420 (95.7) | 2.2 |
|  |  | Yes | 24 (4.6) | 73 (5.2) | - | 21 (4.8) | 19 (4.3) | - |
|  | Collagen disease | No | 513 (98.1) | 1366 (98.2) | 0.9 | 430 (97.9) | 430 (97.9) | 0.0 |
|  |  | Yes | 10 (1.9) | 25 (1.8) | - | 9 (2.1) | 9 (2.1) | - |
|  | Peptic ulcer | No | 493 (94.3) | 1276 (91.7) | 9.9 | 412 (93.8) | 416 (94.8) | 3.9 |
|  |  | Yes | 30 (5.7) | 115 (8.3) | - | 27 (6.2) | 23 (5.2) | - |
|  | Mild liver disease^a^ | No | 491 (93.9) | 1279 (91.9) | 7.5 | 408 (92.9) | 404 (92.0) | 3.5 |
|  |  | Yes | 32 (6.1) | 112 (8.1) | - | 31 (7.1) | 35 (8.0) | - |
|  | Diabetes | No | 453 (86.6) | 1187 (85.3) | 3.7 | 381 (86.8) | 387 (88.2) | 4.1 |
|  |  | Yes | 70 (13.4) | 204 (14.7) | - | 58 (13.2) | 52 (11.8) | - |
|  | Hemiplegia | No | 521 (99.6) | 1389 (99.9) | 4.7 | 438 (99.8) | 438 (99.8) | 0.0 |
|  |  | Yes | 2 (0.4) | 2 (0.1) | - | 1 (0.2) | 1 (0.2) | - |
|  | Renal dysfunction | No | 470 (89.9) | 1251 (89.9) | 0.2 | 394 (89.7) | 398 (90.7) | 3.1 |
|  |  | Yes | 53 (10.1) | 140 (10.1) | - | 45 (10.3) | 41 (9.3) | - |
|  | Diabetes mellitus with chronic complications | No | 501 (95.8) | 1353 (97.3) | 8.1 | 422 (96.1) | 420 (95.7) | 2.3 |
|  |  | Yes | 22 (4.2) | 38 (2.7) | - | 17 (3.9) | 19 (4.3) | - |
|  | Solid cancer, leukemia, lymphoma | No | 353 (67.5) | 1145 (82.3) | 34.7 | 301 (68.6) | 307 (69.9) | 3.0 |
|  |  | Yes | 170 (32.5) | 246 (17.7) | - | 138 (31.4) | 132 (30.1) | - |
|  | Moderate to high liver dysfunction | No | 516 (98.7) | 1361 (97.8) | 6.2 | 432 (98.4) | 431 (98.2) | 1.8 |
|  |  | Yes | 7 (1.3) | 30 (2.2) | - | 7 (1.6) | 8 (1.8) | - |
|  | Metastatic solid tumors | No | 503 (96.2) | 1313 (94.4) | 8.4 | 419 (95.4) | 425 (96.8) | 7.1 |
|  |  | Yes | 20 (3.8) | 78 (5.6) | - | 20 (4.6) | 14 (3.2) | - |
|  | AIDS・HIV | No | 523 (100.0) | 1390 (99.9) | 3.8 | 439 (100.0) | 439 (100.0) | - |
|  |  | Yes | 0 (0.0) | 1 (0.1) | - | 0 (0.0) | 0 (0.0) | - |
| Treatments, n (%) | Ventilators | No | 319 (61.0) | 1091 (78.4) | 38.7 | 291 (66.3) | 302 (68.8) | 5.4 |
|  |  | Yes | 204 (39.0) | 300 (21.6) | - | 148 (33.7) | 137 (31.2) | - |
|  | Dialysis | No | 419 (80.1) | 1258 (90.4) | 29.4 | 367 (83.6) | 374 (85.2) | 4.4 |
|  |  | Yes | 104 (19.9) | 133 (9.6) | - | 72 (16.4) | 65 (14.8) | - |
|  | Extracorporeal membrane oxygenation | No | 514 (98.3) | 1117 (80.3) | 60.8 | 430 (97.9) | 434 (98.9) | 7.3 |
|  |  | Yes | 9 (1.7) | 274 (19.7) | - | 9 (2.1) | 5 (1.1) | - |
|  | Intra-aortic balloon pumping | No | 488 (93.3) | 1295 (93.1) | 0.8 | 408 (92.9) | 419 (95.4) | 10.7 |
|  |  | Yes | 35 (6.7) | 96 (6.9) | - | 31 (7.1) | 20 (4.6) | - |
|  | Central venous catheterization | No | 521 (99.6) | 1389 (99.9) | 4.7 | 438 (99.8) | 438 (99.8) | 0.0 |
|  |  | Yes | 2 (0.4) | 2 (0.1) | - | 1 (0.2) | 1 (0.2) | - |
|  | Pleural effusion | No | 375 (71.7) | 1260 (90.6) | 49.7 | 341 (77.7) | 343 (78.1) | 1.1 |
|  |  | Yes | 148 (28.3) | 131 (9.4) | - | 98 (22.3) | 96 (21.9) | - |
|  | Blood purification therapy | No | 419 (80.1) | 1258 (90.4) | 29.4 | 367 (83.6) | 374 (85.2) | 4.4 |
|  |  | Yes | 104 (19.9) | 133 (9.6) | - | 72 (16.4) | 65 (14.8) | - |
|  | Coagulation blood test | No | 14 (2.7) | 168 (12.1) | 36.6 | 14 (3.2) | 14 (3.2) | 0.0 |
|  |  | Yes | 509 (97.3) | 1223 (87.9) | - | 425 (96.8) | 425 (96.8) | - |
| Transfusion, n (%) | Red blood cell transfusion | No | 330 (63.1) | 720 (51.8) | 23.1 | 273 (62.2) | 272 (62.0) | 0.5 |
|  |  | Yes | 193 (36.9) | 671 (48.2) | - | 166 (37.8) | 167 (38.0) | - |
|  | Whole blood transfusion | No | 523 (100.0) | 1391 (100.0) | - | 439 (100.0) | 439 (100.0) | - |
|  |  | Yes | 0 (0.0) | 0 (0.0) | - | 0 (0.0) | 0 (0.0) | - |
| Number of beds in medical facilities, n (%) | <200 beds |  | 12 (2.3) | 110 (7.9) | 25.7 | 9 (2.1) | 28 (6.4) | 21.7 |
|  | ≥200, <500 |  | 304 (58.1) | 717 (51.5) | 13.3 | 259 (59.0) | 239 (54.4) | 9.2 |
|  | ≥500 |  | 207 (39.6) | 564 (40.5) | 2.0 | 171 (39.0) | 172 (39.2) | 0.5 |
| Platelets  (average^b^) | n |  | 42 | 84 | - | 35 | 35 | - |
|  | Median |  | 9.78 | 14.25 | - | 10.20 | 7.90 | - |
| Creatinine  (average^b^) | n |  | 41 | 84 | - | 34 | 35 | - |
|  | Median |  | 1.040 | 0.850 | - | 1.035 | 0.800 | - |
| Total bilirubin  (average^b^) | n |  | 39 | 79 | - | 32 | 34 | - |
|  | Median |  | 0.560 | 0.660 | - | 0.580 | 0.700 | - |
| Direct bilirubin  (average^b^) | n |  | 27 | 41 | - | 21 | 19 | - |
|  | Median |  | 0.200 | 0.300 | - | 0.200 | 0.300 | - |
| FDP/D-dimer  (average^b^) | n |  | 21 | 27 | - | 16 | 13 | - |
|  | Median |  | 15.000 | 8.600 | - | 14.050 | 11.800 | - |
| Platelets  (minimum^c^) | n |  | 42 | 84 | - | 35 | 35 | - |
|  | Median |  | 9.75 | 14.15 | - | 10.20 | 7.90 | - |
| Creatinine  (minimum^c^) | n |  | 41 | 84 | - | 34 | 35 | - |
|  | Median |  | 1.010 | 0.850 | - | 1.000 | 0.800 | - |
| Total bilirubin  (minimum^c^) | n |  | 39 | 79 | - | 32 | 34 | - |
|  | Median |  | 0.560 | 0.660 | - | 0.580 | 0.700 | - |
| Direct bilirubin  (minimum^c^) | n |  | 27 | 41 | - | 21 | 19 | - |
|  | Median |  | 0.200 | 0.300 | - | 0.200 | 0.300 | - |
| FDP/D-dimer  (minimum^c^) | n |  | 21 | 27 | - | 16 | 13 | - |
|  | Median |  | 12.100 | 8.600 | - | 11.550 | 11.800 | - |
| Platelets  (maximum^d^) | n |  | 42 | 84 | - | 35 | 35 | - |
|  | Median |  | 10.25 | 14.65 | - | 10.30 | 7.90 | - |
| Creatinine  (maximum^d^) | n |  | 41 | 84 | - | 34 | 35 | - |
|  | Median |  | 1.040 | 0.850 | - | 1.035 | 0.800 | - |
| Total bilirubin  (maximum^d^) | n |  | 39 | 79 | - | 32 | 34 | - |
|  | Median |  | 0.600 | 0.660 | - | 0.600 | 0.700 | - |
| Direct bilirubin  (maximum^d^) | n |  | 27 | 41 | - | 21 | 19 | - |
|  | Median |  | 0.200 | 0.300 | - | 0.200 | 0.300 | - |
| FDP/D-dimer  (maximum^d^) | n |  | 21 | 27 | - | 16 | 13 | - |
|  | Median |  | 18.200 | 8.600 | - | 16.600 | 11.800 | - |

^a^Includes diseases that seem to be severe (e.g., liver cirrhosis), but as defined in the previous study (Quan H, Med Care, 2005, 43, 1130)

^b^When there were repeated test results, we adopted the average.

^c^When there were repeated test results, we adopted the minimum.

^d^When there were repeated test results, we adopted the maximum.

rTM, recombinant thrombomodulin; DIC, disseminated intravascular coagulation; FDP, fibrinogen/fibrin degradation products; SD, standard deviation
