# Supplementary material for: Assessment of bleeding in patients with disseminated intravascular coagulation after receiving surgery and recombinant human soluble thrombomodulin: A cohort study using a database
Source: PLoS One. 2018 Oct 8;13(10):e0205146. doi: 10.1371/journal.pone.0205146 (PMC6175500; doi:10.1371/journal.pone.0205146)
Supplement: S6 Table — rTM, recombinant thrombomodulin; CI, confidence interval. (DOCX) [file pone.0205146.s010.docx]

**S6 Table. Bleeding-related adverse events with an incidence >1% in patients undergoing hepatic, biliary, or pancreatic surgery**

| **Bleeding-related adverse events** | **Groups (N=568**  **patients per group)** | **Incidence (%)** | **Risk ratio** | | |
| --- | --- | --- | --- | --- | --- |
|  |  |  | **Point  estimate** | **95% CI** | **p-value** |
|  |  |  |  |  |  |
| All bleeding-related adverse events | non-rTM group | 90 (15.8) | 1.000 | - | 0.1778 |
|  | rTM group | 74 (13.0) | 0.822 | 0.618–1.093 |  |
| Gastrointestinal hemorrhage | non-rTM group | 13 (2.3) | 1.000 | - | 0.8398 |
|  | rTM group | 12 (2.1) | 0.923 | 0.425–2.006 |  |
| Other hemorrhage | non-rTM group | 86 (15.1) | 1.000 | - | 0.0551 |
|  | rTM group | 64 (11.3) | 0.744 | 0.550–1.006 |  |
| Hemorrhagic shock | non-rTM group | 45 (7.9) | 1.000 | - | 0.0153 |
|  | rTM group | 25 (4.4) | 0.556 | 0.346–0.893 |  |
| Hemorrhagic anemia | non-rTM group | 28 (4.9) | 1.000 | - | 0.3085 |
|  | rTM group | 21 (3.7) | 0.750 | 0.431–1.305 |  |
| Postoperative anemia | non-rTM group | 7 (1.2) | 1.000 | - | 0.7805 |
|  | rTM group | 6 (1.1) | 0.857 | 0.290–2.535 |  |
| Acute blood loss anemia | non-rTM group | 4 (0.7) | 1.000 | - | 0.2554 |
|  | rTM group | 8 (1.4) | 2.000 | 0.606–6.604 |  |

rTM, recombinant thrombomodulin; CI, confidence interval
