# Supplementary material for: Assessment of bleeding in patients with disseminated intravascular coagulation after receiving surgery and recombinant human soluble thrombomodulin: A cohort study using a database
Source: PLoS One. 2018 Oct 8;13(10):e0205146. doi: 10.1371/journal.pone.0205146 (PMC6175500; doi:10.1371/journal.pone.0205146)
Supplement: S7 Table — rTM, recombinant thrombomodulin; CI, confidence interval. (DOCX) [file pone.0205146.s011.docx]

**S7 Table. Bleeding-related adverse events with an incidence >1% in patients undergoing other gastrointestinal surgeries**

| **Bleeding-related adverse events** | **Groups (N=614**  **patients per group)** | **Incidence (%)** | **Risk ratio** | | |
| --- | --- | --- | --- | --- | --- |
|  |  |  | **Point  estimate** | **95% CI** | **p-value** |
|  |  |  |  |  |  |
| All bleeding-related adverse events | non-rTM group | 196 (31.9) | 1.000 | - | 0.8061 |
|  | rTM group | 192 (31.3) | 0.980 | 0.831–1.155 |  |
| Gastrointestinal hemorrhage | non-rTM group | 14 (2.3) | 1.000 | - | 0.7118 |
|  | rTM group | 16 (2.6) | 1.143 | 0.563–2.321 |  |
| Wound hemorrhage | non-rTM group | 9 (1.5) | 1.000 | - | 0.8172 |
|  | rTM group | 10 (1.6) | 1.111 | 0.455–2.715 |  |
| Other hemorrhage | non-rTM group | 183 (29.8) | 1.000 | - | 0.4888 |
|  | rTM group | 172 (28.0) | 0.940 | 0.789–1.120 |  |
| Hemorrhagic shock | non-rTM group | 90 (14.7) | 1.000 | - | 0.8074 |
|  | rTM group | 87 (14.2) | 0.967 | 0.736–1.270 |  |
| Hemorrhagic anemia | non-rTM group | 65 (10.6) | 1.000 | - | 0.2076 |
|  | rTM group | 52 (8.5) | 0.800 | 0.565–1.132 |  |
| Postoperative anemia | non-rTM group | 16 (2.6) | 1.000 | - | 0.8557 |
|  | rTM group | 15 (2.4) | 0.938 | 0.468–1.879 |  |
| Acute blood loss anemia | non-rTM group | 8 (1.3) | 1.000 | - | 0.4898 |
|  | rTM group | 11 (1.8) | 1.375 | 0.557–3.395 |  |
| Postoperative hemorrhagic shock | non-rTM group | 9 (1.5) | 1.000 | - | 1.0000 |
|  | rTM group | 9 (1.5) | 1.000 | 0.400–2.502 |  |
| Hemorrhagic trend | non-rTM group | 8 (1.3) | 1.000 | - | 1.0000 |
|  | rTM group | 8 (1.3) | 1.000 | 0.378–2.647 |  |
| Hemorrhage | non-rTM group | 2 (0.3) | 1.000 | - | 0.1774 |
|  | rTM group | 6 (1.0) | 3.000 | 0.608–14.806 |  |

rTM, recombinant thrombomodulin; CI, confidence interval
