# Supplementary material for: Assessment of bleeding in patients with disseminated intravascular coagulation after receiving surgery and recombinant human soluble thrombomodulin: A cohort study using a database
Source: PLoS One. 2018 Oct 8;13(10):e0205146. doi: 10.1371/journal.pone.0205146 (PMC6175500; doi:10.1371/journal.pone.0205146)
Supplement: S8 Table — rTM, recombinant thrombomodulin; CI, confidence interval. (DOCX) [file pone.0205146.s012.docx]

**S8 Table. Bleeding-related adverse events with an incidence >1% in patients undergoing cardiac or cardiovascular surgery**

| **Bleeding-related adverse events** | **Groups (N=439**  **patients per group)** | **Incidence (%)** | **Risk ratio** | | |
| --- | --- | --- | --- | --- | --- |
|  |  |  | **Point  estimate** | **95% CI** | **p-value** |
|  |  |  |  |  |  |
| All bleeding-related adverse events | non-rTM group | 142 (32.3) | 1.000 | - | 0.0003 |
|  | rTM group | 94 (21.4) | 0.662 | 0.529–0.829 |  |
| Intracranial hemorrhage | non-rTM group | 5 (1.1) | 1.000 | - | 0.7619 |
|  | rTM group | 6 (1.4) | 1.200 | 0.369–3.903 |  |
| Gastrointestinal hemorrhage | non-rTM group | 8 (1.8) | 1.000 | - | 0.5913 |
|  | rTM group | 6 (1.4) | 0.750 | 0.262–2.144 |  |
| Subcutaneous/Muscle hemorrhage | non-rTM group | 7 (1.6) | 1.000 | - | 0.5629 |
|  | rTM group | 5 (1.1) | 0.714 | 0.228–2.233 |  |
| Wound hemorrhage | non-rTM group | 10 (2.3) | 1.000 | - | 0.2022 |
|  | rTM group | 5 (1.1) | 0.500 | 0.172–1.451 |  |
| Other hemorrhage | non-rTM group | 119 (27.1) | 1.000 | - | 0.0006 |
|  | rTM group | 76 (17.3) | 0.639 | 0.495–0.825 |  |
| Hemorrhagic shock | non-rTM group | 59 (13.4) | 1.000 | - | 0.0049 |
|  | rTM group | 33 (7.5) | 0.559 | 0.373–0.839 |  |
| Hemorrhagic anemia | non-rTM group | 39 (8.9) | 1.000 | - | 0.3202 |
|  | rTM group | 31 (7.1) | 0.795 | 0.505–1.250 |  |
| Postoperative anemia | non-rTM group | 10 (2.3) | 1.000 | - | 0.3184 |
|  | rTM group | 6 (1.4) | 0.600 | 0.220–1.637 |  |
| Acute blood loss anemia | non-rTM group | 11 (2.5) | 1.000 | - | 0.0260 |
|  | rTM group | 2 (0.5) | 0.182 | 0.041–0.816 |  |
| Postoperative hemorrhagic shock | non-rTM group | 9 (2.1) | 1.000 | - | 0.1744 |
|  | rTM group | 4 (0.9) | 0.444 | 0.138–1.432 |  |
| Acute massive hemorrhage | non-rTM group | 9 (2.1) | 1.000 | - | 0.0535 |
|  | rTM group | 2 (0.5) | 0.222 | 0.048–1.023 |  |
| Abdominal aortic aneurysm rupture | non-rTM group | 6 (1.4) | 1.000 | - | 0.5276 |
|  | rTM group | 4 (0.9) | 0.667 | 0.189–2.346 |  |

rTM, recombinant thrombomodulin; CI, confidence interval
