# Supplementary material for: Assessment of bleeding in patients with disseminated intravascular coagulation after receiving surgery and recombinant human soluble thrombomodulin: A cohort study using a database
Source: PLoS One. 2018 Oct 8;13(10):e0205146. doi: 10.1371/journal.pone.0205146 (PMC6175500; doi:10.1371/journal.pone.0205146)
Supplement: S9 Table — (DOCX) [file pone.0205146.s013.docx]

**S9 Table. Bleeding-related adverse events with an incidence >1% in patients undergoing any type of surgery** **requiring blood transfusion or a hemostatic procedure after the day of DIC treatment**

| **Bleeding-related adverse events** | **Groups (N=2117 patients per group)** | **Incidence (%)** | **Risk ratio** | | |
| --- | --- | --- | --- | --- | --- |
|  |  |  | **Point  estimate** | **95% CI** | **p-value** |
| Intracranial hemorrhage | non-rTM group | 24 (1.1) | 1.000 | - | 0.0070 |
|  | rTM group | 8 (0.4) | 0.333 | 0.150–0.740 |  |
| Respiratory hemorrhage | non-rTM group | 2 (0.1) | 1.000 | - | 0.5713 |
|  | rTM group | 1 (0.0) | 0.500 | 0.045–5.510 |  |
| Gastrointestinal hemorrhage | non-rTM group | 31 (1.5) | 1.000 | - | 0.2751 |
|  | rTM group | 23 (1.1) | 0.742 | 0.434–1.268 |  |
| Other hemorrhage | non-rTM group | 361 (17.1) | 1.000 | - | 0.0003 |
|  | rTM group | 277 (13.1) | 0.767 | 0.664–0.887 |  |
| Hemorrhagic shock | non-rTM group | 181 (8.5) | 1.000 | - | 0.0001 |
|  | rTM group | 117 (5.5) | 0.646 | 0.516–0.809 |  |
| Hemorrhagic anemia | non-rTM group | 132 (6.2) | 1.000 | - | 0.0261 |
|  | rTM group | 99 (4.7) | 0.750 | 0.582–0.966 |  |
| Postoperative anemia | non-rTM group | 31 (1.5) | 1.000 | - | 0.6942 |
|  | rTM group | 28 (1.3) | 0.903 | 0.544–1.500 |  |
| Acute blood loss anemia | non-rTM group | 21 (1.0) | 1.000 | - | 0.8782 |
|  | rTM group | 22 (1.0) | 1.048 | 0.578–1.899 |  |
| Hemorrhagic trend | non-rTM group | 21 (1.0) | 1.000 | - | 0.0198 |
|  | rTM group | 8 (0.4) | 0.381 | 0.169–0.858 |  |

DIC, disseminated intravascular coagulation; rTM, recombinant thrombomodulin; CI, confidence interval
