# Supplementary material for: Assessment of bleeding in patients with disseminated intravascular coagulation after receiving surgery and recombinant human soluble thrombomodulin: A cohort study using a database
Source: PLoS One. 2018 Oct 8;13(10):e0205146. doi: 10.1371/journal.pone.0205146 (PMC6175500; doi:10.1371/journal.pone.0205146)
Supplement: S10 Table — DIC, disseminated intravascular coagulation; rTM, recombinant thrombomodulin; CI, confidence interval. (DOCX) [file pone.0205146.s014.docx]

**S10 Table. Bleeding-related adverse events with an incidence >1% in patients undergoing hepatic, biliary, or pancreatic surgery requiring blood transfusion or a hemostatic procedure after the day of DIC treatment**

| **Bleeding-related adverse events** | **Groups (N=568 patients per group)** | **Incidence (%)** | **Risk ratio** | | |
| --- | --- | --- | --- | --- | --- |
|  |  |  | **Point  estimate** | **95% CI** | **p-value** |
| Gastrointestinal hemorrhage | non-rTM group | 7 (1.2) | 1.000 | - | 0.7951 |
|  | rTM group | 8 (1.4) | 1.143 | 0.417–3.131 |  |
| Other hemorrhage | non-rTM group | 64 (11.3) | 1.000 | - | 0.2839 |
|  | rTM group | 53 (9.3) | 0.828 | 0.587–1.169 |  |
| Hemorrhagic shock | non-rTM group | 29 (5.1) | 1.000 | - | 0.1436 |
|  | rTM group | 19 (3.3) | 0.655 | 0.372–1.155 |  |
| Hemorrhagic anemia | non-rTM group | 25 (4.4) | 1.000 | - | 0.1560 |
|  | rTM group | 16 (2.8) | 0.640 | 0.345–1.186 |  |
| Postoperative anemia | non-rTM group | 7 (1.2) | 1.000 | - | 0.7805 |
|  | rTM group | 6 (1.1) | 0.857 | 0.290–2.535 |  |
| Acute blood loss anemia | non-rTM group | 4 (0.7) | 1.000 | - | 0.2554 |
|  | rTM group | 8 (1.4) | 2.000 | 0.606–6.604 |  |

DIC, disseminated intravascular coagulation; rTM, recombinant thrombomodulin; CI, confidence interval
