# Supplementary material for: Assessment of bleeding in patients with disseminated intravascular coagulation after receiving surgery and recombinant human soluble thrombomodulin: A cohort study using a database
Source: PLoS One. 2018 Oct 8;13(10):e0205146. doi: 10.1371/journal.pone.0205146 (PMC6175500; doi:10.1371/journal.pone.0205146)
Supplement: S11 Table — DIC, disseminated intravascular coagulation; rTM, recombinant thrombomodulin; CI, confidence interval. (DOCX) [file pone.0205146.s015.docx]

**S11 Table. Bleeding-related adverse events with an incidence >1% in patients undergoing gastrointestinal surgeries requiring blood transfusion or a hemostatic procedure after the day of DIC treatment**

| **Bleeding-related adverse events** | **Groups (N=614 patients per group)** | **Incidence (%)** | **Risk ratio** | | |
| --- | --- | --- | --- | --- | --- |
|  |  |  | **Point  estimate** | **95% CI** | **p-value** |
| Gastrointestinal hemorrhage | non-rTM group | 12 (2.0) | 1.000 | - | 0.8333 |
|  | rTM group | 11 (1.8) | 0.917 | 0.408–2.062 |  |
| Wound hemorrhage | non-rTM group | 8 (1.3) | 1.000 | - | 0.8072 |
|  | rTM group | 9 (1.5) | 1.125 | 0.437–2.897 |  |
| Other hemorrhage | non-rTM group | 147 (23.9) | 1.000 | - | 0.4563 |
|  | rTM group | 136 (22.1) | 0.925 | 0.754–1.135 |  |
| Hemorrhagic shock | non-rTM group | 61 (9.9) | 1.000 | - | 0.4059 |
|  | rTM group | 70 (11.4) | 1.148 | 0.829–1.588 |  |
| Hemorrhagic anemia | non-rTM group | 61 (9.9) | 1.000 | - | 0.0845 |
|  | rTM group | 44 (7.2) | 0.721 | 0.498–1.045 |  |
| Postoperative anemia | non-rTM group | 16 (2.6) | 1.000 | - | 0.4461 |
|  | rTM group | 12 (2.0) | 0.750 | 0.358–1.572 |  |
| Postoperative hemorrhagic shock | non-rTM group | 8 (1.3) | 1.000 | - | 1.0000 |
|  | rTM group | 8 (1.3) | 1.000 | 0.378–2.647 |  |
| Acute blood loss anemia | non-rTM group | 7 (1.1) | 1.000 | - | 0.7952 |
|  | rTM group | 8 (1.3) | 1.143 | 0.417–3.132 |  |
| Hemorrhagic trend | non-rTM group | 8 (1.3) | 1.000 | - | 0.5922 |
|  | rTM group | 6 (1.0) | 0.750 | 0.262–2.149 |  |

DIC, disseminated intravascular coagulation; rTM, recombinant thrombomodulin; CI, confidence interval
