# Supplementary material for: Assessment of bleeding in patients with disseminated intravascular coagulation after receiving surgery and recombinant human soluble thrombomodulin: A cohort study using a database
Source: PLoS One. 2018 Oct 8;13(10):e0205146. doi: 10.1371/journal.pone.0205146 (PMC6175500; doi:10.1371/journal.pone.0205146)
Supplement: S12 Table — DIC, disseminated intravascular coagulation; rTM, recombinant thrombomodulin; CI, confidence interval. (DOCX) [file pone.0205146.s016.docx]

**S12 Table. Bleeding-related adverse events with an incidence >1% in patients undergoing cardiac or cardiovascular surgery requiring blood transfusion or a hemostatic procedure after the day of DIC treatment**

| **Bleeding-related adverse events** | **Groups (N=439 patients per group)** | **Incidence (%)** | **Risk ratio** | | |
| --- | --- | --- | --- | --- | --- |
|  |  |  | **Point  estimate** | **95% CI** | **p-value** |
| Intracranial hemorrhage | non-rTM group | 5 (1.1) | 1.000 | - | 1.0000 |
|  | rTM group | 5 (1.1) | 1.000 | 0.292–3.430 |  |
| Gastrointestinal hemorrhage | non-rTM group | 6 (1.4) | 1.000 | - | 0.7619 |
|  | rTM group | 5 (1.1) | 0.833 | 0.256–2.710 |  |
| Subcutaneous/muscle hemorrhage | non-rTM group | 6 (1.4) | 1.000 | - | 0.7619 |
|  | rTM group | 5 (1.1) | 0.833 | 0.256–2.710 |  |
| Wound hemorrhage | non-rTM group | 9 (2.1) | 1.000 | - | 0.2884 |
|  | rTM group | 5 (1.1) | 0.556 | 0.188–1.644 |  |
| Other hemorrhage | non-rTM group | 113 (25.7) | 1.000 | - | 0.0001 |
|  | rTM group | 66 (15.0) | 0.584 | 0.444–0.768 |  |
| Hemorrhagic shock | non-rTM group | 55 (12.5) | 1.000 | - | 0.0024 |
|  | rTM group | 28 (6.4) | 0.509 | 0.329–0.787 |  |
| Hemorrhagic anemia | non-rTM group | 38 (8.7) | 1.000 | - | 0.2027 |
|  | rTM group | 28 (6.4) | 0.737 | 0.461–1.179 |  |
| Postoperative anemia | non-rTM group | 10 (2.3) | 1.000 | - | 0.2022 |
|  | rTM group | 5 (1.1) | 0.500 | 0.172–1.451 |  |
| Acute blood loss anemia | non-rTM group | 11 (2.5) | 1.000 | - | 0.0260 |
|  | rTM group | 2 (0.5) | 0.182 | 0.041–0.816 |  |
| Postoperative hemorrhagic shock | non-rTM group | 8 (1.8) | 1.000 | - | 0.2548 |
|  | rTM group | 4 (0.9) | 0.500 | 0.152–1.648 |  |
| Acute massive hemorrhage | non-rTM group | 9 (2.1) | 1.000 | - | 0.0535 |
|  | rTM group | 2 (0.5) | 0.222 | 0.048–1.023 |  |
| Abdominal aortic aneurysm rupture | non-rTM group | 6 (1.4) | 1.000 | - | 0.3247 |
|  | rTM group | 3 (0.7) | 0.500 | 0.126–1.987 |  |

DIC, disseminated intravascular coagulation; rTM, recombinant thrombomodulin; CI, confidence interval
